# *Nb*-FAR-1: A key developmental protein affects lipid droplet accumulation and cuticle formation in *Nippostrongylus brasiliensis*

**DOI:** 10.1371/journal.pntd.0012769

**Published:** 2025-01-17

**Authors:** Wenmin Qi, Fei Shen, Chuyue Wang, Juan Wen, Xi Pan, Zhongying Zhao, Lihua Xiao, Yaoyu Feng, Dongjuan Yuan

**Affiliations:** 1 State Key Laboratory for Animal Disease Control and Prevention, Center for Emerging and Zoonotic Diseases, College of Veterinary Medicine, South China Agricultural University, Guangzhou, Guangdong, China; 2 Department of Biology, Hong Kong Baptist University, Hong Kong, China; National Institutes of Allergy and Infectious Diseases, NIH, UNITED STATES OF AMERICA

## Abstract

Fatty acid and retinol binding proteins (FARs) are lipid-binding protein that may be associated with modulating nematode pathogenicity to their hosts. However, the functional mechanism of FARs remains elusive. We attempt to study the function of a certain FAR that may be important in the development of *Nippostrongylus brasiliensis*. *Nb*-FAR-1 was highly expressed throughout developmental stages by RNA-seq data and qPCR analyses, and *Nb*-FAR-1 was a secretory protein and abundant in the excretory-secretory products. *Nb*-FAR-1 could bind fatty acids and retinol. Fatty acid pattern of parasitic adults was more similar to rat intestine than to free-living L3s, indicating that *N*. *brasiliensis* may be dependent on the host to obtain fatty acids. Lentivirus-mediated RNAi was performed on L3s, resulting in a reduction in the expression of *Nb*-*far-1* gene. Furthermore, these RNAi effects could be maintained in several generations. The offspring L3s in *Nb*-*far-1* RNAi group had a reduction in lipid droplets within the subcuticle and the swelling of the perioral epidermis, accompanied with down-regulated expression of enzymes in amino acid and glycerolipid metabolism and glycometabolism for growth by RNA-seq data. Adults in *Nb*-*far-1* RNAi group had the crumpled epidermis loosely attached to the basal membrane of body surface and the breakage of mouth epidermis, accompanied with a decrease in adult egg-shedding and an appearance of abnormal eggs. *In vitro* culture of eggs showed decreased efficiency of egg hatchability and larval development in the *Nb*-*far-1* RNAi group. Transcriptomic analysis showed that interference with *Nb*-*far-1* expression induced downregulated expression of major sperm protein and serpin for reproduction, and collagen for epidermis formation in adults, most of which were relatively high expression in adults but low expression in L3s in the WT group. Thus, *Nb*-FAR-1 may affect the reproduction, growth, and development of *N*. *brasiliensis* by regulating the level of lipids.

## Author summary

Fatty acid and retinol-binding protein 1 (FAR-1) is lipid-binding protein that may be associated with modulation of host immune responses and provide insights into nematode pathogenicity to their hosts. However, the role of FAR-1 protein on parasitic nematodes remains unclear. *Nippostrongylus brasiliensis* was employed to identify the characteristics of *Nb*-*far-1* gene, its expression patterns, ligand binding properties and the functions associated with it. The secretory *Nb*-FAR-1 protein was found to be highly expressed in *N*. *brasiliensis* ESPs and demonstrated affinity for fatty acids and retinol. Interference with *Nb*-*far-1* gene expression resulted in a reduction in the formation of lipid droplets in L3s, a decrease in a rate of adult egg-shedding, egg hatchability, and larval development. Therefore, we proposed that *N*. *brasiliensis Nb*-FAR-1 protein may serve as a causative agent in nematode growth, development, and reproduction by regulating the level of lipids.

## Introduction

Parasitic nematodes have fewer orthologs in fatty acid biosynthetic and metabolic pathways than free-living *Caenorhabditis elegans* [1, 2] and rely on lipid-binding and transport proteins to uptake, transport, and phagocytose various lipids or metabolites from their hosts. Fatty acid and retinol-binding proteins (FARs) are nematode-specific proteins with the ability to bind fatty acids and retinol and promote the uptake, transport, and distribution of lipids and retinol [3]. FAR members have the genus-level richness in parasitic nematodes of *Meloidogyne* (1–3), *Globodera* (2–4), *Steinernema* (37–43), *Strongyloides* (16), filarial species (1–3), *Ancylostoma* (18–30), and have low sequence identity [4]. Thus, it is speculated that FARs have undergone multiple expansions and divergences to adapt to the parasitism in the plants, invertebrates, and vertebrates across nematode lineages [4].

All FARs have a typical Gp-FAR-1 domain (pfam05823), thereby the first reported member of the family was directly named FAR-1 in some nematodes. The reported FAR-1 proteins show functional divergence in biological processes. *Onchocerca volvulus* FAR-1 has been proposed to deplete retinol at the parasite site to induce the skin and eye pathology of river blindness [5, 6]. The root-knot nematode *Meloidogyne javanica* FAR-1 can modulate host gene expression to influence host immune responses [7]. Interference with *Globodera pallida far-1* expression affected plant lipoxygenase-mediated defense signaling and may interact with eicosanoids to sequester host retinoids for immune evasion [8]. *Radopholus similis* FAR-1 inhibited the expression levels of allene oxide synthase in the jasmonic acid pathway, thus playing a critical role in plant defense response [9]. *Steinernema carpocapsae* FAR-1 suppressed fly immunity, resulting in increased host susceptibility to bacterial infection via the phenol oxidase cascade and antimicrobial peptide production [10]. Thus, these reported FAR-1 may be associated with modulation of host immune responses and provide insights into nematode parasitism, but the role of FAR-1 protein on nematodes remains elusive. To elucidate the characteristics of the FAR-1 protein in the family, a previous study had designated the member with the highest sequence identity to Gp-FAR-1 as FAR-1 by searching the genomes of 58 nematodes. These FAR-1 proteins have the high sequence similarity and shared high expression levels in infective third-stage larvae (iL3s), fourth-stage larvae (L4s), and adults of *Strongyloides* and members of Strongylida [4].

Infective larvae of *Nippostrongylus brasiliensis* have a migration route to the lungs and intestines of rodents via subcutaneous injection, which is similar to that of hookworms and has been useful in modeling some aspects of nematode development and host immunopathology [11–13]. *N*. *brasiliensis* has 12 FAR proteins, and we attempt to study a certain FAR that may be important in development. Although *N*. *brasiliensis Nb*-FAR-1 shares a high degree of sequence identity with other nematodes [4], the expression pattern across developmental stages and other characterizations of *Nb*-FAR-1 remain elusive. Therefore, we studied genomic characteristics and expression patterns of 12 *far* genes and then focused on one FAR that may be important in development by genome-wide analysis. Hence, we elucidated the ligand-binding properties and function of this FAR in *N*. *brasiliensis* development and reproduction.

## Material and methods

### Ethics statement

Sprague-Dawley (SD) rats were obtained from the Guangdong Experimental Animal Center. All animal procedures conformed to the Chinese National Institute of Health Guide for the Care and Use of Laboratory Animals, and the protocol was approved by the South China Agricultural University Committee for Animal Research with approval number 2023F187.

### Worm collection and culture

*N*. *brasiliensis* eggs in rat feces were cultured on the moistened filter paper with 1% penicillin-streptomycin-amphotericin B solution and incubated at 26°C in the dark. First-stage larvae (L1s), second-stage larvae (L2s), and L3s were collected after 24 hours (h), 48 h, and 7 days of culture, respectively. The iL3 were used to infect SD rats by subcutaneous injection in the abdominal skin. L4s were collected from rat lungs at 3–4 days post-infection (dpi), and L5s and adult worms were collected from rat intestines at 6 dpi and 12 dpi, respectively. To study egg hatchability and larval development, fifty adult worms were incubated for 30 minutes (min) at 37°C in 500 μL phosphate-buffered saline (PBS) buffer to lay eggs. L1s, L2s and L3s were collected after 24 h, 48 h, and 72 h of incubation of eggs in PBS buffer at 37°C under 5% CO_2_, respectively. Worm morphology was observed by stereomicroscopy (Leica, Germany), and the size of eggs, larvae, and adults was calculated using Image J.

### Fatty acid methylation and gas chromatography (GC) analysis

*N*. *brasiliensis* adult worms were mainly found to be present in the region from the posterior of the duodenum to the anterior of the jejunum of rat intestines at 11 dpi. Rat intestinal tissues in this region were collected and the contents removed. Heptadecanoic acid (C17:0) (5 mg/mL, 10 μL) was added as an internal standard to 30 mg dry tissue of L3s, adults, and rat intestinal tissues in each tube. Fatty acids were methyl esterified using 2 mL 5% sulfuric acid/methanol solution and 300 μL methylbenzene at 95°C for 1.5 h. Fatty acids were extracted from the supernatant using 2 mL 0.9% NaCl solution and 1 mL n-hexane by centrifugation at 5,000 rpm for 5 min. Fatty acid methyl esters were separated on an Agilent 7890A GC equipped with a flame ionization detector (FID) and a fused silica capillary column (DB-Fast FAME, Agilent, CA). The initial temperature was 80°C and held for 0.5 min, ramped to 165°C at 40°C/min and held for 1 min, then ramped to 230°C at 4°C/min and held for 6 min. Fatty acid peaks were identified by comparing the retention time of each peak with validated fatty acid standards (Supelco, Bellefonte, PA). Unidentified peaks were not included in the calculation of total fatty acid percentages. Total fatty acid concentration (nmol/g viscera) was calculated by comparing GC peak areas relative to that of C17:0 internal standard [14]. Fatty acid percentages were expressed as % of the total fatty acids in each sample. The percentage of fatty acids was calculated using the formula: fatty acid % = (S1/S2)×N/M*100%, where S1: total peak area of fatty acids, S2: peak area of C17:0, N: the content of C17:0 in the sample, M: the content of the sample.

### Cloning, expression and purification of recombinant protein

Total RNA was extracted from adults and reverse transcribed to cDNA using the PrimeScript RT Reagent Kit (TaKaRa, Japan) according to the manufacturer’s protocols after removing contaminating genomic DNA with DNase I (Sigma, USA). The cDNA encoding *N*. *brasiliensis Nb*-FAR-1 protein was amplified using the primers of 5’- CGCGGATCCAGCCCGATCAGTAGCATC -3’ and 5’- CCGCTCGAGGTTCTTAGCCAACAGTGT -3’ and Phanta Max Super-Fidelity DNA Polymerase (Vazyme, China). The *Nb*-*far-1* gene was cloned into plasmid pET-28a with His tag using *BamH* I and *Xho* I restriction enzymes (S1B Fig). Recombinant *Nb*-FAR-1 protein with His tag was expressed in *Escherichia coli* Transetta (DE3) under 1 mM isopropylthio-β-galactoside (IPTG) at 16°C for 18 h. Recombinant *Nb*-FAR-1 protein was purified by chromatography column packed with HisSep Ni-NTA agarose resin (Yeasen, China). Purified recombinant *Nb*-FAR-1 protein was obtained and concentrated using a 3 kilodalton (kDa) ultrafiltration tube (Merck Millipore, Germany). The purified *Nb*-FAR-1 protein was analyzed by 12.5% sodium dodecyl sulfate—polyacrylamide gel electrophoresis (SDS-PAGE). Protein concentrations were determined using Enhanced BCA Protein Assay Kit (Beyotime, Shanghai, China).

### Fluorescence-based ligand binding assays

The fatty acid and retinol binding activities of recombinant *N*. *brasiliensis Nb*-FAR-1 protein were measured using the fluorescent analog 11-(dansylamino) undecanoic acid (DAUDA) (Sigma, USA) as described previously [4]. DAUDA, retinol (Sigma, USA), and other fatty acids (Aladdin, Shanghai) were prepared as a stock solution of 10 mM in ethanol and stored at -20°C. The Kd value of *Nb*-FAR-1 protein with DAUDA was estimated using the reaction of 10 μM DAUDA and 0.5 μM, 1 μM, 2.5 μM, 5 μM, 10 μM, 12.5 μM, 15 μM, 20 μM, 25 μM, 30 μM, 35 μM, and 40 μM *Nb*-FAR-1 protein, respectively. The Kd value of *Nb*-FAR-1 protein with retinol was detected using the reaction of 5 μM *Nb*-FAR-1 protein and 1 μM, 5 μM, 10 μM, 15 μM, 20 μM, 25 μM, 30 μM, 35 μM and 40 μM of retinol, respectively. The binding ability of *Nb*-FAR-1 protein with fatty acids was detected using the reaction of 10 μM DAUDA and 1 μM, 2.5 μM, 5 μM, 10 μM, and 50 μM fatty acids (C16:0, palmitic acid; C18:0, stearic acid; C18:1, oleic acid; C18:2, linoleic acid; C20:4, arachidonic acid; C20:5, eicosapentaenoic acid), respectively. Fluorescence emission spectra for *Nb*-FAR-1 bound to DAUDA or retinol were recorded at 25°C with a total volume of 100 μL per well in a 96-well black microplate (Corning, USA) using a SpectraMax M5 (Molecular Devices, USA). The excitation wavelengths used for DAUDA and retinol were 345 nm and 350 nm, respectively. The Kd value for *Nb*-FAR-1 protein binding to DAUDA or retinol was calculated by GraphPad Prism 9.0 using this formula: Y = Bmax*X / (Kd + X), where X: the concentration of DAUDA or retinol, Y: the fluorescence value measured after binding of *Nb*-FAR-1 to DAUDA or retinol, Kd: the equilibrium dissociation constant, in the same units as X. It is the fluorescent ligand concentration needed to achieve a half-maximum binding at equilibrium, Bmax: the maximum specific binding in the same units as Y. Competition binding experiments for *Nb*-FAR-1 bound to fatty acids were performed as previously described [4, 15]. Relative fluorescence intensity in each fatty acid group was calculated by comparing the peak fluorescence intensity relative to that of the group of DAUDA bound to *Nb*-FAR-1 protein.

### Preparation and mass spectrometry analysis of adult excretory-secretory products (ESPs)

Adults were collected from the intestine of infected SD rats at 11 dpi. Adult worms were washed, and 100 adults each were cultured in 1 mL culture medium (RPMI1640, 1% glucose, 100 U/mL penicillin, 100 μg/mL streptomycin and 50 μg/mL gentamicin) for 24 h at 37°C with 5% CO_2_ as previously reported [16]. Approximately 6000 adults were incubated in the culture medium, and the culture medium supernatant was collected. The supernatant was centrifuged at 2,000 relative centrifugal force (rcf) for 10 min at 4°C and filtered by 0.22 μm membrane filtration (Biosharp, China). The 700 μg ESPs were obtained from the supernatant, which was concentrated by a 3 kDa ultrafiltration tube (Merck Millipore, Germany) and then replaced with PBS buffer and protease inhibitor. The ESPs were revealed by silver staining, and the protein bands were mainly concentrated at 15 kDa, 25 kDa, and 45–60 kDa (S2 Fig). ESPs were performed on the Easy nLC 1200 system (ThermoFisher) equipped with an analytical column (Acclaim PepMap RSLC, 75 μm × 25 cm C18-2 μm 100 Å) for chromatographic separation. Mass spectrometry detection was performed on a Q Exactive mass spectrometer (ThermoFisher, USA) equipped with a Nano Flex ion source (Wininnovate Bio, Shenzhen, China). Liquid chromatography and tandem mass spectrometry (LC-MS/MS) analysis revealed a total of 895 *N*. *brasiliensis* proteins in adult ESPs, and 573 proteins were identified as reliable proteins (unique peptides ≥ 2).

### Identification of small interfering RNA (siRNA)

Four siRNAs were biosynthesized by GenePharma (Suzhou, China). Adults were used to evaluate the effects of siRNAs on *far-1* gene expression. Adults were divided into six groups: (i) wild-type (WT), (ii) 0.2 mM siRNA negative control (NC), (iii) 0.2 mM siRNA-*far-1*-583, (iv) 0.2 mM siRNA-*far*-1-651, (v) 0.2 mM siRNA-*far*-1-310, and (vi) 0.2 mM siRNA-*far-1*-517. After 24 h of incubation with siRNAs, fifty adults in each group were washed three times with PBS buffer and harvested for quantitative real-time PCR (qRT-PCR).

### Lentivirus far-1-651 and far-1-310 administration

Target gene-specific hairpin stem sequences were composed of siRNA sense sequences, loop sequences, siRNA antisense sequences, and polyA. Sense and antisense sequences of siRNA-*Nb*-*far-1*-651 and siRNA-*Nb*-*far-1*-310 were amplified from *N*. *brasiliensis* cDNA by PCR using OneTaq polymerase (New England Biolabs, USA). The shRNA sequences were as follows: shRNA-*Nb*-*far-1*-651 sequences 5’-GACATACTCTCATTTGTATTTCAAGAGAATACAAATGAGAGTATGTCTTTTTT-3’ and shRNA-*Nb*-*far-1*-310 sequences 5’- GCTTTCGCCAAGGAGATCATTCAAGAGATGATCTCCTTGGCGAAAGCTTTTTT-3’. These sequences were cloned into the virus-encoding plasmid pGIPZ under the transcript of human miRNA30 and designated as miRNA-adapted shRNA (shRNAmir) [17]. The primers of shRNAmir (5’-CAAGCCCGGTGCCTGAGTT-3’ and 5’-TGGCCGGCCGCATTAGTCTT-3’) were used to identify the shRNAmir sequences in the vectors of pGIPZ-*Nb*-*far-1*-651 and pGIPZ-*Nb*-*far-1*-310. Plasmids psPAX_2_ and pMD2.G were obtained from Didier Trono (Addgene plasmids # 12260 and 12259, respectively). Approximately 2.5 × 10^6^ HEK293T cells maintained in Dulbecco’s modified Eagle’s medium (DMEM) at 37°C, 10% fetal calf serum (FCS), 2 mM L-glutamine, 100 U/mL penicillin and 100 μg/mL streptomycin (Sigma, USA) were transfected with 2.5 μg pGIPZ-*Nb*-*far-1*-651/310, 2.5 μg psPAX_2_ and 1 μg pMD2.G using Lipofectamine 2000 (Life Technologies, USA). The transfection medium was replaced with 2 mL culture medium containing 5% FCS, 20 mM HEPES (Sigma, USA) and 10 μM cholesterol (balanced with methyl-β-cyclodextrin, Sigma, USA) after 16 h. VSV-G-pseudotyped, replication-incompetent lentivirus (LV) particles in the cell supernatant were harvested after an additional 48 h incubation at 37°C and 10% CO_2_. The supernatants were centrifuged at 1,000 × g for 10 min at 4°C, passed through a 0.45 μm Acrodisc syringe filter (Macklin, China), and stored at -80°C in 1 mL aliquots. Functional virus titers were estimated as TCID50 (50% tissue culture infectious dose) using the Reed-Muench method.

### Parasite infection, recovery and transduction with lentivirus

*N*. *brasiliensis* was maintained in female SD rats, and iL3s were isolated and washed extensively in wash buffer (PBS buffer, 0.45% glucose, 100 U/mL penicillin, 100 μg/mL streptomycin, 100 μg/mL gentamicin) by centrifugation at 150 g for 3 minutes. Larvae were activated in worm culture medium (RPMI1640 with 2% FBS, 1.5% glucose, 2 mM L-glutamine, 100 U/mL penicillin, 100 μg/mL streptomycin, 100 μg/mL gentamicin, 20 mM HEPES) for 48 to 72 h and then washed twice in serum-free medium before exposure to LV. Approximately 2,000 activated L3s were exposed to LV at multiplicities of infection (MOIs) of 200 and 400 in 1 mL of serum-free medium containing 10 μg/mL polybrene (Sigma, USA). Controls were unexposed L3s (wild type, WT) and L3s exposed to LV lacking a small hairpin structure (empty vector, EV). LV-containing culture medium for L3s was replaced with culture medium after 24 h of incubation, and interference effects of L3s were evaluated after an additional 48 h incubation. Approximately 2000 L3s were used to infect 7-week-old female SD rats by subcutaneous injection, and adults were harvested at 12 dpi. Rat lung and intestine tissues were prefixed in 4% paraformaldehyde for hematoxylin and eosin (H&E) staining to analyze the pathological changes of rat intestine after *N*. *brasiliensis* infection. L3s were collected and prefixed in 1% paraformaldehyde, dehydrated in 60% isopropanol for 2 min, and stained with isopropyl alcohol Oil Red O solution (1% Triton X-100, 30 min).

### Reverse transcription PCR (RT-PCR) and real-time quantitative PCR (RT-qPCR)

Total RNA was extracted from recombinant LV using Simply P Total RNA Extraction Kit (BioFlux, USA) and total RNA was extracted from L3s using Trizol (Invitrogen, USA). Total RNA was reverse transcribed to cDNA using PrimeScript RT Reagent Kit (Takara, Japan) according to the manufacturer’s protocols after removing contaminating genomic DNA with DNase I (Sigma, USA). Total DNA from L3s with LV treatment was extracted using DNeasy Blood & Tissue Kit (Qiagen, Germany). The PCR amplification of shRNAmir sequence from the cDNA of recombinant LV and total DNA of L3s was performed using One Taq DNA polymerase (New England Biolabs, USA) and the primers of shRNAmir. The qPCR amplification of *Nb*-*far-1* gene in L3s was performed using SYBR Green Real-Time PCR Master Mix (Toyobo, Japan) on a Light Cycler 480 Instrument II (Roche, Switzerland). The primers for qPCR of *Nb*-*far-1* gene were 5’-GTTCTTAGCCAACAGTGTCTC-3’ and 5’-GGTAACAAGCCAAACCTCG-3’. The primers for the *gapdh* internal control gene were 5’-GCAGCAGACGGACCAATGAAGG-3’ and 5’-CACGAAGTTAGGGTTGAGCGAGATG-3’.

### Scanning electron microscope (SEM) and transmission electron microscope (TEM)

For SEM observation, the worms were prefixed in 2.5% glutaraldehyde, postfixed in 1% OsO_4_, dehydrated through a graded series of ethanol (30%, 50%, 70%, 80%, 90%, and 100%), and then dehydrated in a Leica model CDP 300 critical point dryer with liquid CO_2_. The samples were coated with platinum-palladium in an ACE 600 ion sputter (Leica, Germany) and observed in an EVO MA 15 (ZEISS, Germany). For TEM observation, the worms were prefixed in 2.5% glutaraldehyde, uranyl acetate, dehydrated through a graded series of ethanol (30%, 50%, 70%, 85%, 95%, and 100%), permeated and embedded with acetone resin mixtures in different proportions (3,1, 1:1, 1:3, and 0:1). The samples were sectioned after drying and observed in model Talos F200S (Thermo Fisher Scientific, USA).

### Expression profile analyses

Total RNA was extracted from *N*. *brasiliensis* eggs, L1s, L2s, L3s, L4s, L5s, and adults using Trizol (Invitrogen, USA) to determine expression pattern of *Nb*-*far-1* gene across developmental stages. Total RNA was extracted from *N*. *brasiliensis* L3s and adult worms with interfering *far-1* expression using Trizol (Invitrogen, USA). Total RNA was used for transcriptome sequencing using the Illumina NovaSeq 6000 high-throughput sequencing platform (Personalbio, Shanghai). RNA-seq data of *N*. *brasiliensis* across developmental stages and L3s and adult worms with interfering *Nb*-*far-1* expression were obtained and were deposited in the SRA database under accession number: PRJNA1159469 and PRJNA1156889, respectively. FastQC (v0.11.6) was used for quality control, and Trim-galore (v0.6.6) was used to filter out low quality reads. Reads were mapped to the reference genome (GenBank accession: GCA_030553155.1) using HISAT2 (v2.1) [18]. HTSeq (0.9.1) was used to count the reads on each gene as the raw expression level. Gene transcription was then normalized by calculating the FPKM for each gene based on the gene length and the number of reads mapped to that gene. DESeq in R software was used to analyze differential expression of genes. DEGs in L3s and adults were selected based on |log1.5 Fold Change|>1 and *P* <0.05. GO terms and KEGG pathway analyses were performed using topGO and clusterProfiler (3.4.4), respectively. The TBtools-II (v2.085) was used to visualize the expression level of genes in the heatmap [19].

### Statistical analysis

Data were expressed as mean ± SD (standard deviation). One-way ANOVA and t-test analyses were used to evaluate differences in the experiments. *P* < 0.05 was considered statistically significant. Statistical analysis was performed using Prism 8.0 (GraphPad Software, CA).

## Results

### *N*. *brasiliensis far* genes are diverse in sequence identity and expression pattern

**Fig 1:**
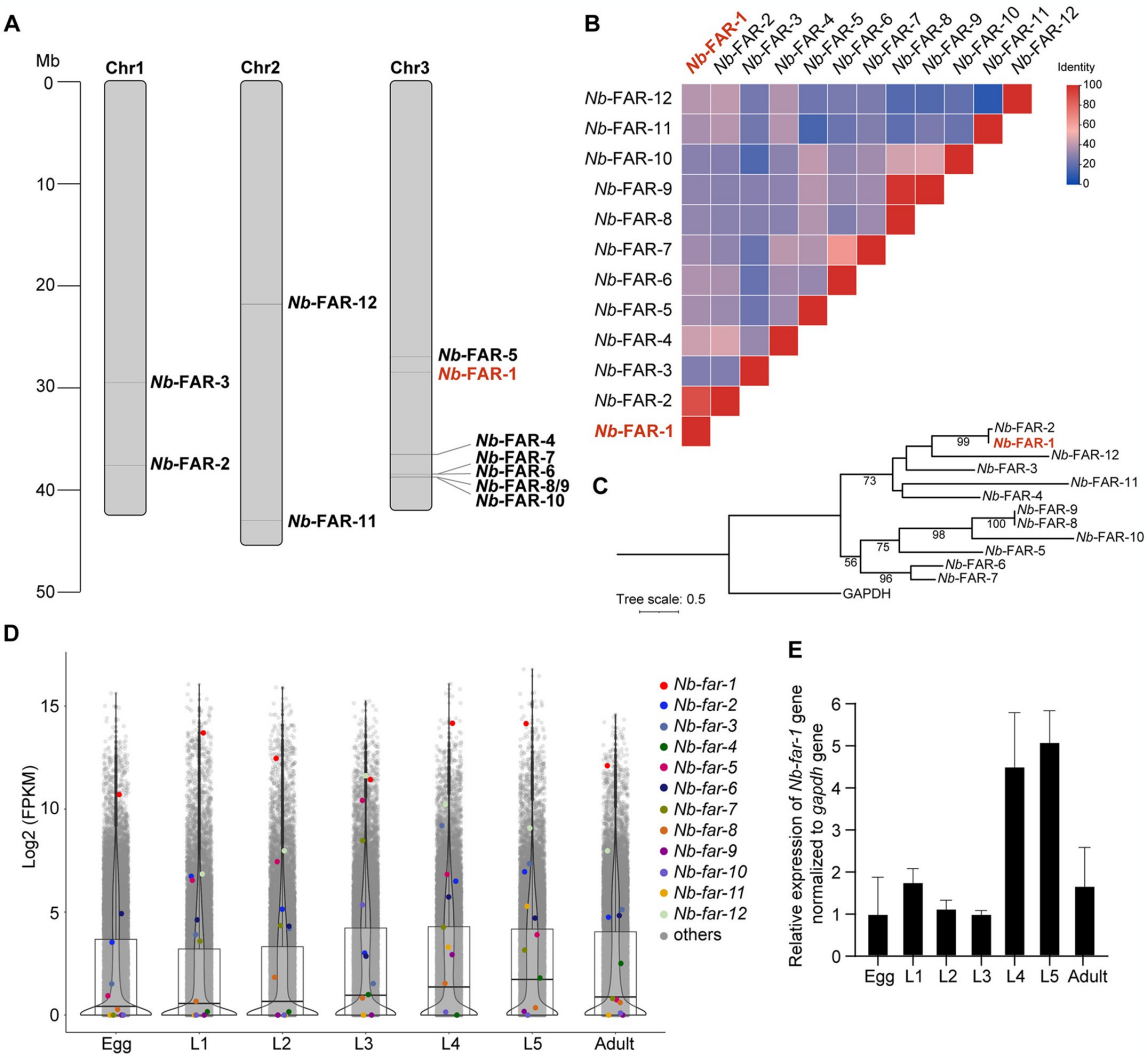
Sequence features and expression pattern of 12 *far* genes in *N*. *brasiliensis*. (A) Location of *far* genes on the chromosome of *N*. *brasiliensis*. Detailed location information was shown in S1 Table. (B) Sequence identity analysis of 12 FAR proteins in *N*. *brasiliensis*. (C) Maximum likelihood protein phylogenetic tree of 12 FAR proteins from *N*. *brasiliensis*. Bootstrap values were shown in the nodes. The scale bar represents the number of amino acid substitutions per site. (D) Expression pattern of *far* genes across developmental stages of *N*. *brasiliensis* using RNA-seq data. Detailed expression values were shown in S2 Table. (E) Expression pattern of the *Nb*-*far-1* gene across developmental stages of *N*. *brasiliensis* by qPCR (n = 3).

*N*. *brasiliensis far* genes are located on chromosomes I, II, III, among which *Nb*-*far*-2 and *Nb*-*far*-3 genes are distributed on chromosome I, *Nb*-*far*-11 and *Nb*-*far*-12 genes are distributed on chromosome II, and other 8 *far* genes are distributed on chromosome III (Fig 1A and S1 Table). *Nb*-*far*-8 and *Nb*-*far*-9 genes are located at the same site on chromosome III and belong to different transcripts (Fig 1A and S1 Table). Except for *Nb*-FAR-2, *Nb*-FAR-1 had low sequence identity with other *Nb*-FARs (Fig 1B and 1C), but expression profiling showed that *Nb*-*far-2* had a low expression level and only *Nb*-*far-1* had a high expression level across developmental stages (Fig 1D and 1E and S2 Table).

### Secretory *Nb*-FAR-1 protein was abundant in *N*. *brasiliensis* ESPs

**Fig 2:**
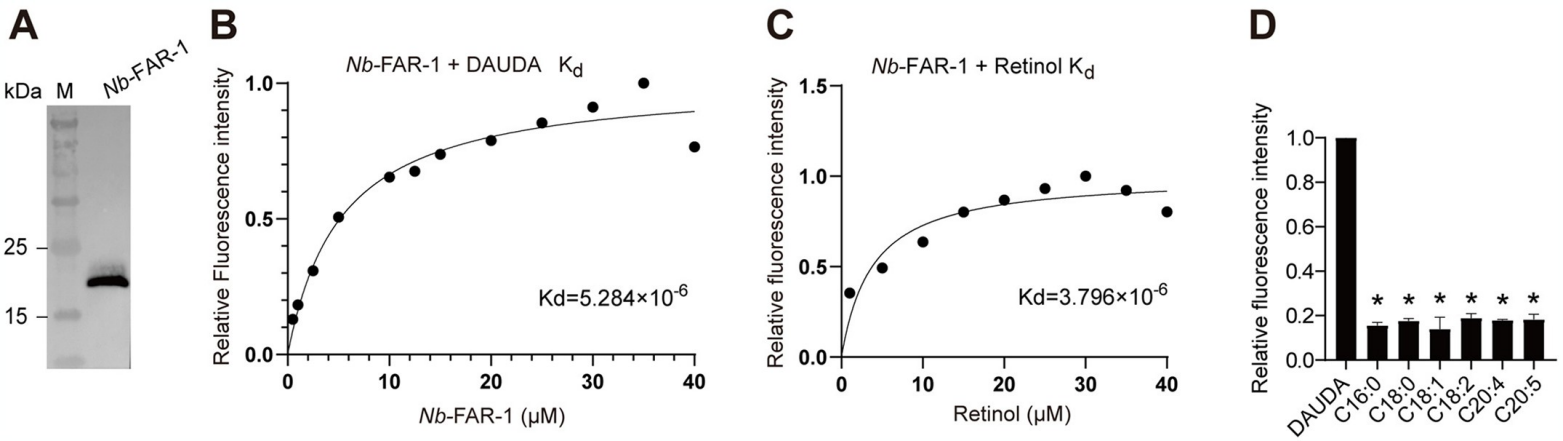
Analysis of the binding activities of *Nb*-FAR-1 protein with different fatty acids and retinol. (A) Prokaryotic expression and purification of recombinant *Nb*-FAR-1 protein. (B) Change in relative fluorescence intensity of DAUDA (10 μM) in the presence of increasing concentrations of *Nb*-FAR-1 protein. The best fit curve was used to determine the equilibrium dissociation constant (Kd) for the DAUDA: *Nb*-FAR-1 interaction. (C) Similar analysis of binding affinity and Kd calculation for the retinol: *Nb*-FAR-1 interaction. (D) Competition binding effect of a 5-fold excess of unlabeled fatty acids with different carbon chain lengths and number of double bonds on the fluorescence intensity of DAUDA-*Nb*-FAR-1 complex (measured at 380~700 nm). * *p* <0.0001 means difference in mean fluorescence intensity compared to the DAUDA-*Nb*-FAR-1 control group (n = 3). C16:0, palmitic acid; C18:0, stearic acid; C18:1, oleic acid; C18:2, linoleic acid; C20:4, arachidonic acid; C20:5, eicosapentaenoic acid.

We cloned the *N*. *brasiliensis Nb*-*far-1* gene (NCBI accession: PP197658) with a length of 3,175 bp, including 5’ untranslated region (UTR), coding sequence (CDS), and 3’ UTR (S1A Fig). The CDS region of *Nb*-*far-1* gene is 543 bp long and encodes a protein of 180 amino acids with approximately 20 kDa (Figs 2A and S1B). *Nb*-FAR-1 has a hydrophobic signal peptide of 16 amino acids at N-terminus with predication by SignalP. *Nb*-FAR-1 occupied the top 73 abundant proteins in the mass spectrometric detection of ESPs by LC-MS/MS (S2 Fig and S3 Table). Thus, secretory *Nb*-FAR-1 is the high expression protein in the ESPs of *N*. *brasiliensis*.

### *Nb*-FAR-1 had binding affinities with fatty acids and retinol

As for substrate binding, fluorescence-based ligand binding analysis showed that recombinant *Nb*-FAR-1 could bind to fluorescent fatty acid analog DAUDA with a Kd value of 5.284×10^−6^ (Fig 2A and 2B). Naturally fluorescent retinol could bind to *Nb*-FAR-1 with a Kd value of 3.796×10^−6^ (Fig 2C). The decrease in fluorescence intensity was observed with addition of fatty acids in the solution containing *Nb*-FAR-1 and DAUDA compared to *Nb*-FAR-1+DAUDA group. The decrease in relative fluorescence intensity could be observed with an increase in the concentration of fatty acids of C16:0, C18:0, C18:1, C18:2, C20:4, and C20:5 (Figs 2D and S3A–S3F), indicating that *Nb*-FAR-1 had the binding ability with fatty acids, as described for FAR-1 from other species [4, 20]. Thus, *N*. *brasiliensis Nb*-FAR-1 had the functions of holding fatty acids and retinol.

### Fatty acid pattern in adult *N*. *brasiliensis* were similar to rat intestines

*N*. *brasiliensis* had the abundant fatty acids in free-living L3s and parasitic adults. The content of fatty acids in L3s, adults and rat intestines reached to 83.1±1.2 mg/g, 44.2±7.0 mg/g, 76.7±12.1 mg/g in dry tissues, respectively. The percentages of saturated fatty acids (SFAs) and unsaturated fatty acids (UFAs) in L3s were 15.1% and 84.9%, respectively, and the main UFAs included myristoleic acid (C14:1), hexadecadienoic acid (C16:2), C18:1, C18:2, dihomo-γ-linolenic acid (C20:3, DGLA), C20:4, and C20:5 (Table 1). The percentages of SFAs and UFAs in adults were 44.1% and 55.9%, respectively, which were similar to 43.4% and 56.6% in rat intestine. The main fatty acids in adults and rat intestine were C16:0, C18:0, C18:1, C18:2, and C20:4, and the levels of C16:0, C18:0, and C18:2 in adults were more similar to rat intestine than L3s (Table 1). The content of PUFAs in the parasitic stage of *N*. *brasiliensis* may be influenced by the host. The preference of *Nb*-FAR-1 for fatty acids was further investigated by adding fatty acids of different chain lengths in the DAUDA assay. The ratio of the peak values of fluorescence intensity between DAUDA+*Nb*-FAR-1+50 μM fatty acid group and DAUDA+*Nb*-FAR-1 group was used to calculate the binding ability of *Nb*-FAR-1 and fatty acids. The results showed that the fluorescence intensity decreased by approximately 80% compared to control group DAUDA+*Nb*-FAR-1 (S3A–S3F Fig). The high DAUDA displacement occurred with fatty acids of C16:0, C18:0, C18:1, C18:2, C20:4, and C20:5 (Fig 2D). Thus, the fatty acid patterns of *N*. *brasiliensis* in the parasitic adults are similar to those in the rat intestine, suggesting that *N*. *brasiliensis* may take up exogenous fatty acids from the diet or the host.

**Table 1:** The compositions of fatty acids in *N*. *brasiliensis* and rat intestine parasitized with adult worms (%, n = 4).

Fatty acids	L3s	Adults	Rat intestine
C14:0	0.9±0.1	0.7±0.2	0.5±0.1
C14:1	15.9±0.2^#^	4.4±0.9*	0.0±0.0
C15:0	0.6±0.0	0.4±0.2	0.2±0.0
C15:1	2.3±0.2^#^	0.0±0.0*	0.4±0.1
C16:0	4.1±0.1^#^	19.1±4.0	20.1±0.9
C16:1	1.5±0.1	1.8±0.4	1.2±0.5
C16:2n-6	4.9±0.0^#^	1.2±0.2*	0.0±0.0
C17:1	2.7±0.0^#^	0.9±0.2*	0.0±0.0
C18:0	8.2±0.1^#^	23.7±3.0	19.0±1.3
C18:1n-9c	24.9±0.2	23.6±3.1*	16.2±2.5
C18:2n-6c	9.8±0.1	14.8±4.6	16.7±1.6
C18:3n-6	1.0±0.0^#^	0.3±0.3	0.1±0.0
C18:3n-3	0.3±0.0^#^	0.0±0.0*	0.3±0.1
C20:0	0.5±0.0	0.2±0.4*	1.3±0.2
C20:1	1.2±0.0^#^	1.0±0.0	0.7±0.2
C20:2	1.3±0.1	1.2±0.4	0.8±0.1
C20:3n-6	4.7±0.0^#^	1.3±0.9	1.4±0.2
C20:4n-6(AA)	6.7±0.1	4.8±4.7*	14.1±2.5
C22:0	0.8±0.0^#^	0.0±0.0*	1.1±0.1
C20:5n-3(EPA)	7.6±0.1^#^	0.1±0.3	0.3±0.0
C22:1	0.2±0.0^#^	0.4±0.1*	0.2±0.0
C22:6n-3(DHA)	0.0±0.0	0.0±0.0*	2.6±0.8
C24:0	0.0±0.0	0.0±0.0*	1.2±0.2
C24:1	0.0±0.0	0.0±0.0*	1.4±0.3
SFAs	15.1	44.1	43.4
UFAs	84.9	55.9	56.6
PUFAs	36.3	23.7	36.3
n-6 PUFAs	28.4	23.6	33.1
n-3 PUFAs	7.9	0.1	3.2

Note:

#: compared with adult worms, *p*<0.05;

*: compared with rat intestine, *p*<0.05.

### LV-mediated RNAi of *Nb*-*far-1* expression affected lipid droplet formation

**Fig 3:**
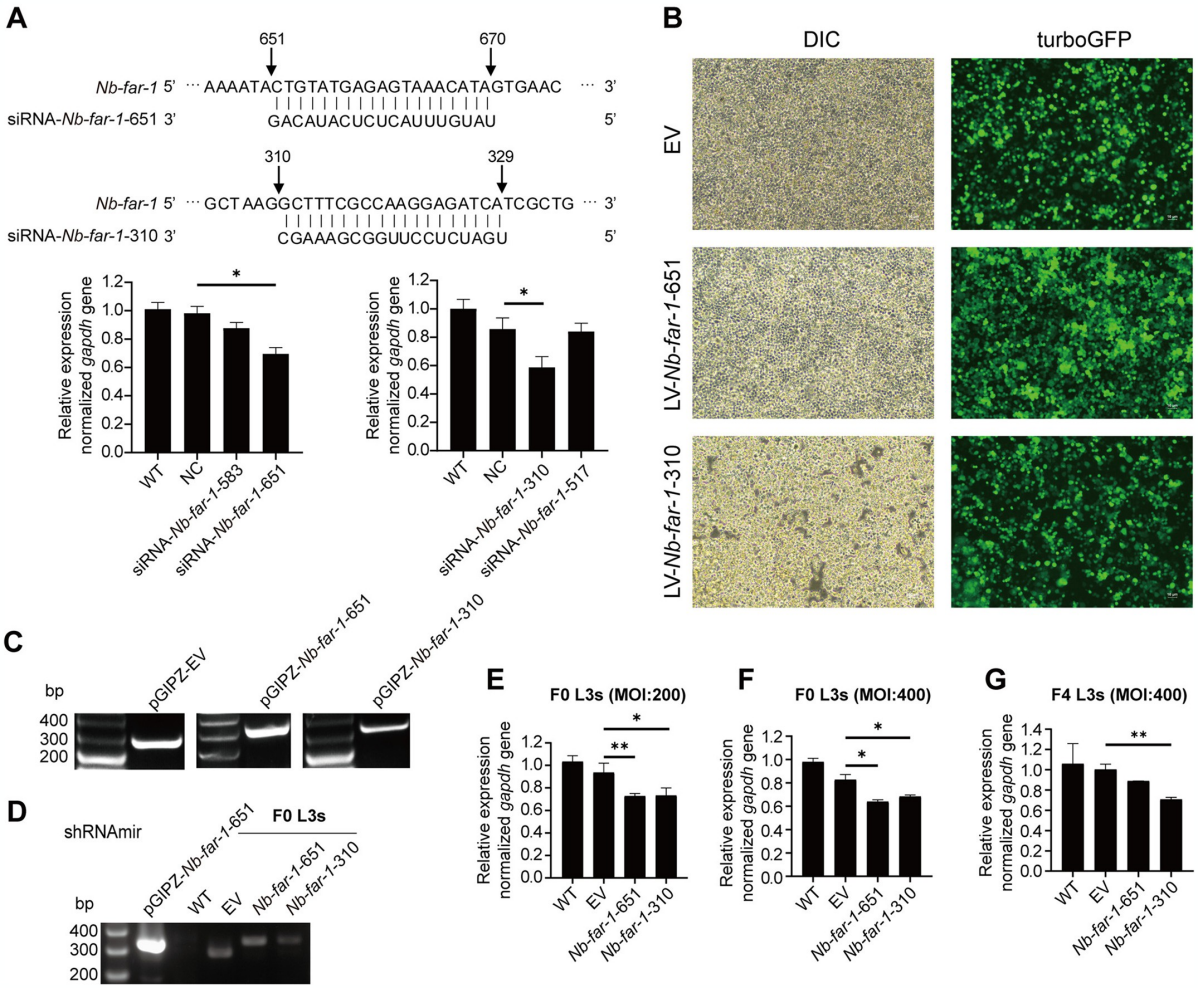
Identification of lentivirus mediated RNAi effects on *Nb*-*far*-*1* expression. (A) Identification of RNAi effects of four siRNAs on L3s by qPCR analysis and alignment of the nucleotide sequences of siRNA-*Nb*-*far-1*-651 and siRNA-*Nb*-*far-1*-310 with *Nb*-*far-1* gene. (B) Lentiviral packaging plasmids were transfected and expressed in 293T cells. (C) PCR identification of shRNAmir-*Nb*-*far-1*-651 and shRNAmir-*Nb*-*far-1*-310 in the lentivirus from the supernatant of 293T cells. (D) PCR analysis of total DNA to identify shRNAmir-*Nb*-*far-1*-651 and shRNAmir-*Nb*-*far-1*-310 integrated into F0 of L3s. (E-F) *Nb*-*far-1* gene transcription in F0 of L3s treated with lentivirus LV-*Nb*-*far-1*-651 and LV-*Nb*-*far-1*-310 at MOI of 200 and 400 by qPCR analysis. (G) *Nb*-*far-1* gene transcription in F4 of L3s after lentivirus treatment by qPCR analysis. WT: wild type; NC: non-specific control siRNA; siRNA-*Nb*-*far-1*-651 and siRNA-*Nb*-*far-1*-310: the positions of the siRNAs on the *Nb*-*far*-1 gene. Data were mean ± SD of three independent experiments (n = 3). *p < 0.05, **p < 0.005.

The effects of four siRNAs on *Nb*-*far-1* expression were evaluated by adding these siRNAs to the culture medium of L3s (Fig 3A). The qPCR assay showed that siRNA-*Nb*-*far-1*-651 and siRNA- *Nb*-*far-1*-310 could downregulate the expression of *Nb*-*far-1* gene by approximately 30% (Fig 3A). Recombinant LV with shRNAmir-*Nb*-*far-1*-651 and shRNAmir-*Nb*-*far-1*-310 as well as empty LV were obtained by packaging in 293T cells (Fig 3B). ShRNAmir was cloned into the vector pGIPZ at the position between mir30a’ and mir30b’ [21, 22], and mir30a’-shRNAmir-mir30b’ was transcribed with the size of 300–400 bp. Mir30a’-shRNAmir-mir30b’ was identified from total RNA of recombinant LV-*Nb*-*far-1*-651 and LV-*Nb*-*far-1*-310 (Fig 3C) and L3s (Fig 3D). No fragment could be amplified from WT group, a fragment of 200–300 bp was identified in EV group, the fragments of 300–400 bp were identified in *Nb*-*far-1*-651 and *Nb*-*far-1*-310 groups from total DNA of L3s after incubation with recombinant LV (Fig 3D).

**Fig 4:**
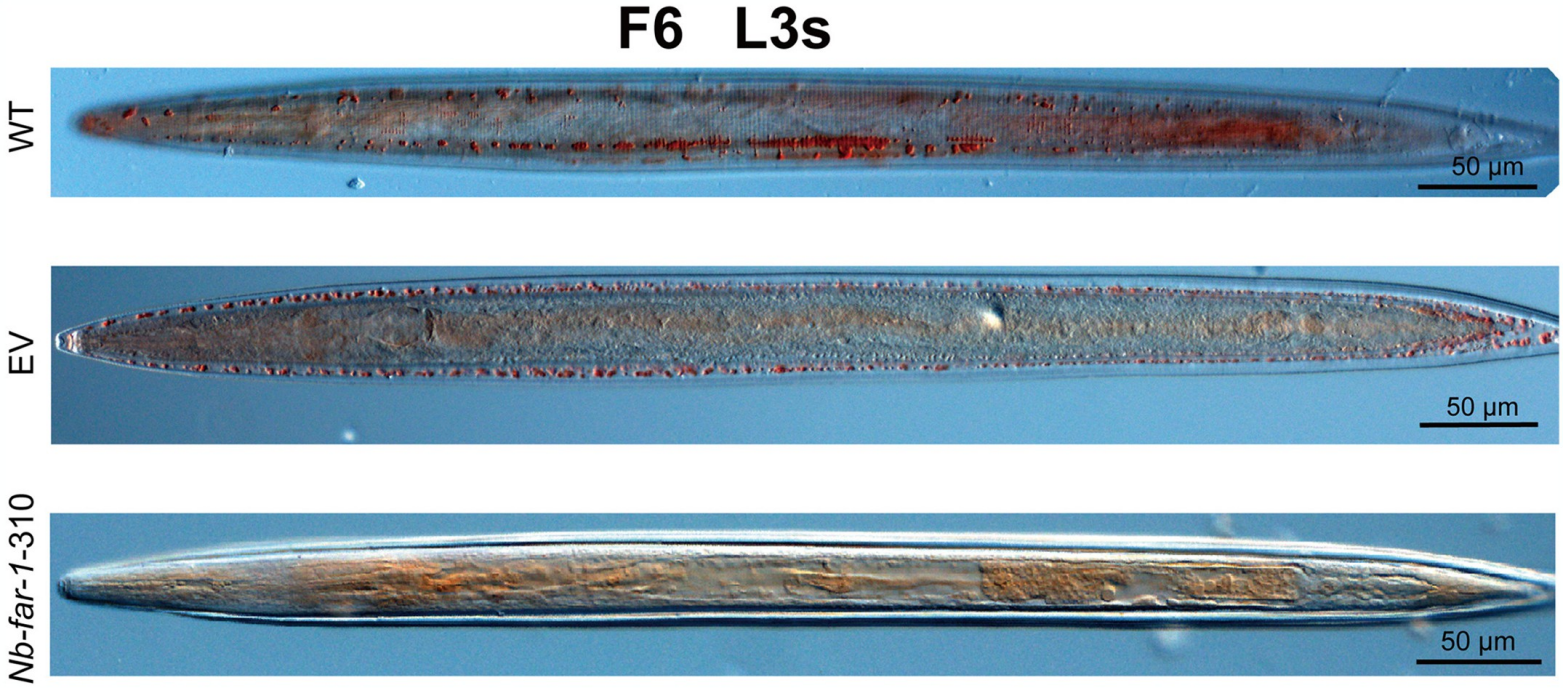
Oil Red O staining of lipid droplets in L3 *N*. *brasiliensis* with LV mediated *Nb*-*far-1* RNAi.

Incubation of LV-*Nb*-*far-1*-651 and LV-*Nb*-*far-1*-310 with F0 generation of L3s could effectively interfere with *Nb*-*far-1* expression by approximately 15.00% at MOI of 200 (Fig 3E) and approximately 20.00% at MOI of 400 compared to EV group (Fig 3F). The offspring of F4 L3s in *Nb*-*far-1*-651 group and *Nb*-*far-1*-310 group also showed the interference effects and down-regulated the expression of *Nb*-*far-1* gene by 11.21% and 29.40%, respectively (Fig 3G). The offspring of F15 L3s showed no interference effects in *Nb*-*far-1*-651 group, but 27.10% in *Nb*-*far-1*-310 group. Thus, the *Nb*-*far-1*-310 group was selected for further analysis. Oil Red O staining of the offspring of L3s showed a significant reduction of lipid droplets on both sides of the subcuticle in *Nb*-*far-1*-310 group compared with EV group (Fig 4). Interference of *Nb*-*far-1* expression may affect lipid transport from the host.

### LV-mediated RNAi of *Nb*-*far-1* gene affected worm gene expression

Transcriptome sequencing was performed on adults of F3 offspring and L3s of F4 offspring to analyze the effects of *Nb*-*far-1* interference at the molecular level (S4 Table). The Adult_310 group had 137 up-regulated DEGs and 369 down-regulated DEGs compared to the Adult_EV group (S4A and S4B Fig). DEGs in Adult_310 group compared to Adult_EV group mainly included *collagen* genes in molting cycle and epidermis formation; growth and development genes for nervous system morphogenesis, tracheal pit formation; growth and development genes in Foxo, EGFR, Wnt and mTOR signaling pathways; metabolic genes in glycometabolism, glycerophospholipid metabolism; genes in ribosome biogenesis by GO and KEGG analyses (S4C and S4D Fig). The results showed that interference with *Nb*-*far-1* gene expression induced down-regulated expression of genes involved in epidermal formation, growth, development, glycometabolism, and glycerophospholipid metabolism.

The L3_310 group had 198 up-regulated DEGs and 561 down-regulated DEGs compared to the L3_EV group (S5A and S5B Fig). DEGs in L3_310 group compared to L3_EV group mainly included genes in glycometabolism and glycerolipid metabolism; amino acid transmembrane transporter, fatty acid and retinoid binding, lysosome, embryonic skeletal joint development, ErbB signaling pathway by GO and KEGG analyses (S5C and S5D Fig). The results showed that interference of *Nb*-*far-1* gene expression caused down-regulated expression of genes in metabolic enzymes, amino acid transmembrane transporter activities, fatty acid and retinol binding in L3s.

### LV-mediated RNAi of *Nb*-*far-1* expression impeded adult egg-shedding and larval development

**Fig 5:**
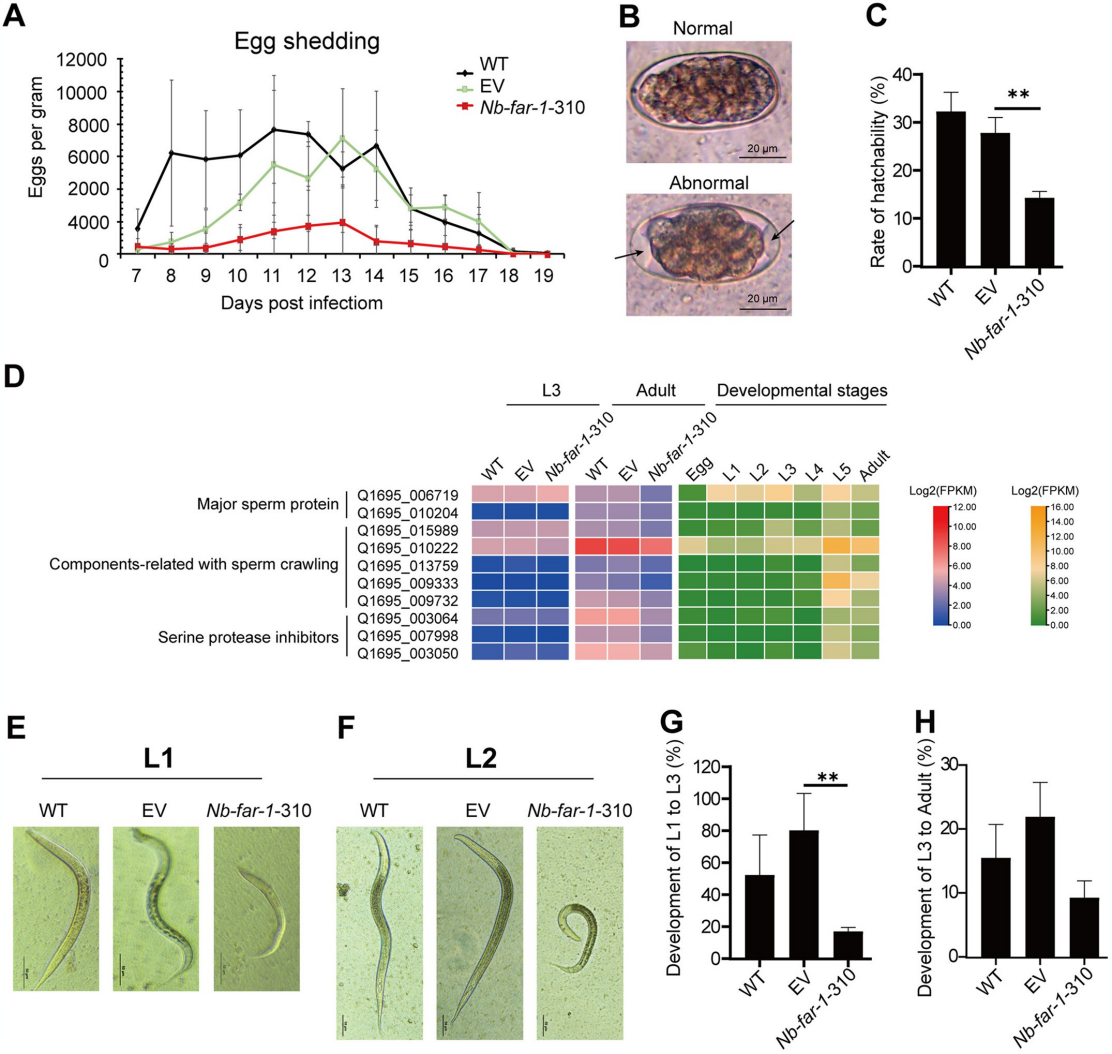
Effects of lentivirus-mediated siRNA of *N*. *brasiliensis Nb*-*far*-*1* gene on female egg-shedding and larval development after treatment with lentivirus LV-*Nb*-*far-1*-310. (A) Effects of *Nb*-*far-1* RNAi on the egg-shedding curve of adult female worms (n = 3). (B) Effects of *Nb*-*far-1* RNAi on the morphology of fecal eggs. Arrow indicates the increased space between blastomere and egg shell. (C) Effects of *Nb*-*far-1* RNAi on changes in egg hatchability (n = 3). (D) Expression pattern of sperm-related genes in *N*. *brasiliensis* with *Nb*-*far-1* RNAi and across developmental stages with RNA-seq data, respectively. (E-F) Effects of *Nb*-*far-1* RNAi on the morphological changes of L1s and L2s. (G) Effects of *Nb*-*far-1* RNAi on the changes of larval development from L1s to L3s (n = 3). (H) Effects of *Nb*-*far-1* RNAi on the changes of larval development from L3s to adults (n = 3). **p < 0.01.

The body weight and the pathological changes (DPI 12) of SD rats infected with 2,000 L3s did not differ significantly among WT, EV, and *Nb*-*far-1*-310 groups, as shown in S6 Fig. Egg-shedding of adult worms was significantly reduced in infected rats, and the egg-shedding period was significantly shortened from 12 days to 9 days (Fig 5A). Some eggs with decreased size and increased space between blastomere and egg shell appeared at 11 dpi in *Nb*-*far-1*-310 group and 14–16 dpi in WT and EV groups (Figs 5B and S7A). *In vitro* culture of eggs showed the decrease in the rates of egg hatchability in *Nb*-*far-1*-310 group compared to WT and EV groups (Fig 5C). Transcriptomic data showed that the expression level of genes in major sperm protein, component associated with sperm crawling, and serine protease inhibitor were down-regulated in *Nb*-*far-1*-310 group compared to WT and EV groups (Fig 5D and S5 Table). Thus, interference of *Nb*-*far-1* expression affected the reproduction of *N*. *brasiliensis*.

*In vitro* culture showed that the developmental rate of larvae was significantly decreased in *Nb*-*far-1*-310 group compared to WT and EV groups (Fig 5E–5G). The length and width of L1s and L2s were decreased in *Nb*-*far-1*-310 group compared to WT and EV groups (S7B and S7C Fig). The molting rate of L3s in *Nb*-*far-1*-310 groups after 8 days of *in vitro* culture was significantly higher than that of WT and EV groups, indicating that the molting of L3s in *Nb*-*far-1*-310 group in advance (S7D Fig). In the parasitic stage, the developmental rate of infective L3s to adults showed the decrease, but it was not statistically significant (Fig 5H). Transcriptomic data showed that the expression level of genes in amino acid metabolism, glycometabolism, lipid metabolism was down-regulated (S8 Fig), which may be related to the dysplasia of larvae. Thus, suppressing of *Nb*-*far-1* gene expression could effectively affect adult egg-shedding, egg hatching, and larval development.

### LV-mediated RNAi of *far-1* gene affected epidermal formation

**Fig 6:**
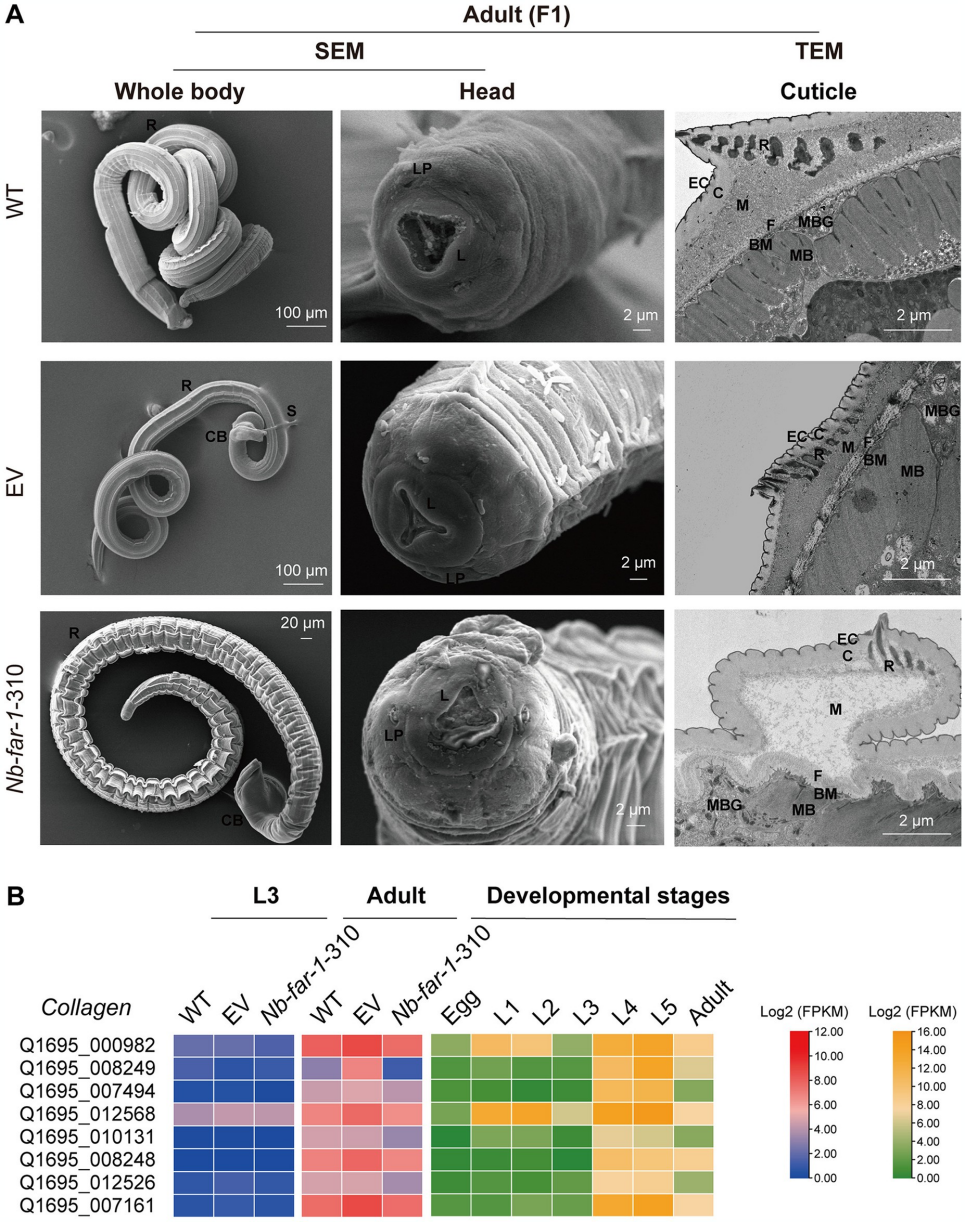
Interference with *Nb*-*far-1* gene expression impeded cuticle formation in *N*. *brasiliensis* after treatment with lentivirus LV-*Nb*-*far-1*-310. (A) SEM and TEM observation of the morphological changes in adult (F1 generation) *N*. *brasiliensis*. L: lip, LP: labial papilla, R: ridge, EC: epicuticle, C: cortical, M: medial, F: fiber, BM: basal membrane, MB: muscle bundle, MBG: muscle bundle gap. (B) Expression pattern of *collagen* in *N*. *brasiliensis* with *Nb*-*far-1* RNAi and across developmental stages with RNA-seq data, respectively.

SEM observation of the epidermis revealed the shrinked body and perioral swelling of L3s (S9 Fig), and the crumpled epidermis loosely attached to the basal membrane in adults with breakage of the mouth epidermis in *Nb*-*far-1*-310 group compared to WT and EV groups (Fig 6). TEM observation showed that the cuticle of adults was composed of the cortical, fiber, and medial layers. The medial layer connected the cortical and fiber layers. The degradation of the medial layer and the loose connection of the cortical and fiber layers were observed in adults of the *Nb*-*far-1*-310 group. Transcriptomic data showed that the gene transcription level of *collagen* was down-regulated in *Nb*-*far-1*-310 group (S7 Table), indicating that interference with *Nb*-*far-1* gene expression could significantly affect the formation or maintenance of the epidermis.

## Discussion

FAR-1 is a nematode-specific protein with the ability to bind fatty acids and retinol. Secretory FAR-1 may be the small molecule released by parasitic nematodes to modulate host lipid pathways, influence host immune responses, and affect nematode parasitism, but the effects of FAR-1 on the nematodes remain elusive.

### Lipid transfer protein *Nb*-FAR-1 might be involved in lipids acquirement for *N*. *brasiliensis*

Parasitic nematodes are generally considered to be deficient in some enzymes involved in lipid synthesis and metabolism and are dependent on their hosts for essential lipids. In this study, the percentages of the main fatty acids C14:1, C16:2, C20:5, C16:0, C18:0, and C18:2 in adult *N*. *brasiliensis* were closer to that of rat small intestine and significantly different from that of free-living L3s, which was consistent with the fatty acids of *D*. *viviparus* in free-living and parasitic stages [23]. Nematodes in parasitic stages can take up fatty acids from their hosts via lipid-binding proteins. The *N*. *brasiliensis* lipid-binding protein *Nb*-FAR-1 had binding properties for C16:0, C18:0, C18:2, C20:5, and retinol. *C*. *elegans* FAR-1 and *A*. *cantonensis* FAR-1 also had lipid-binding activities [4, 24]. However, members of the FAR family differ in their ability to bind ligands. *C*. *elegans* FAR-7 and *A*. *cantonensis* FAR-3 had low lipid-binding abilities, which may be due to the smaller ligand-binding cavities of *C*. *elegans* FAR-7 and *A*. *cantonensis* FAR-3 compared to FAR-1 [4, 24] or other factors such as the charge distribution of the walls in the cavity and the presence of appropriate amino acids to bind a ligand.

*N*. *brasiliensis Nb*-*far-1* was highly expressed across developmental stages compared to other *Nb*-*far* genes, which was consistent with *far-1* in other nematodes [4]. However, *Nb*-*far-1* gene transcription was significantly down-regulated in adults compared to L4 and L5 larvae according to the expression pattern of RNA-seq data and qPCR analysis across developmental stages, which was even reduced by approximately 50% (Fig 1D and 1E). RNAi of *Nb*-*far-1* gene could effectively reduce *Nb*-*far-1* gene expression by approximately 30% in L3s, but no significant decrease in the expression of genes in fatty acid and retinol binding in adults. We proposed that the down-regulated expression of *Nb*-*far-1* gene caused by RNAi in adults might be attenuated by the down-regulated *Nb*-*far-1* gene in adults compared to larvae in the developmental process. Nematode *Nb*-*far-1* was mainly expressed in the hypodermis of *R*. *similis*, *Aphelenchoides besseyi*, *G*. *pallida*, *Pratylenchus penetrans*, *Heterodera avenae*, and *Heterodera filipjevi* [8, 9, 25–27], *and in the* ovaries and testes, muscle layer, intestine, and egg of *Aphelenchoides* species [27, 28]. Lipid droplets of L3s in *N*. *brasiliensis* were distributed in the near-epidermal region and were significantly reduced by interference of *Nb*-*far-1* gene expression. The expression of *C*. *elegans* FAR-7 in the whole hypodermis during starvation suggests an up-regulation to mobilize the greatest amounts of fatty acids necessary for nematode survival [29]. Thus, the *N*. *brasiliensis* lipid-binding protein *Nb*-FAR-1 may be essential for nematode development via fatty acid acquisition.

### *Nb*-FAR-1 regulates the reproduction of adult *N*. *brasiliensis*

Suppression of *Nb*-*far-1* gene expression could reduce reproduction, accompanied by a decrease in adult egg-shedding and an increase in abnormal eggs in *N*. *brasiliensis*. Suppression of *Nb*-*far-1* gene expression induced the downregulation of the reproduction-related genes of MSP and serpin. MSP, the core component in the formation of sperm retractile motility, regulated the motility of nematode spermatocytes [30] and functioned as a hormone to promote oocyte maturation [31]. MSP was a signaling molecule with bidirectional functions of oocyte maturation and sheath contraction. Serpin was a secretory protein of nematode spermatozoa and has an important effect on sperm activation [32]. UFAs were the main components of human spermatozoa, accounting for approximately 50% of total fatty acids, and also play an important role in sperm formation and maintenance of sperm viability [33–35]. UFAs were also required for spermatogenesis in *C*. *elegans* [36]. Thus, the *Nb*-FAR-1 protein affects lipid formation and thus sperm quality, and functions in reproduction and embryonic development of the worm.

### *Nb*-FAR-1 modulates the larval development of *N*. *brasiliensis*

Interference with *Nb*-*far-1* expression significantly affected the growth and development of *N*. *brasiliensis*, with a decrease in egg hatchability and larval growth to the next stages using *in vitro* culture, accompanied by smaller size of eggs, L1s and L2s. Fatty acids and retinols were required for the growth, development and embryogenesis of parasitic nematodes via gene activation, cell signaling pathway, tissue differentiation and repair [27, 37, 38]. Vitamin A (retinol and other related compounds) deficiency in cotton rats would retard embryogenesis in female *Litomosoides carinii* [39]. *In vitro* studies have shown that synthetic retinoids can reduce motility and inhibit the release of microfilariae [40] and molting of larval stages [38]. L3s of *A*. *cantonensis* use ingested exogenous fatty acids to synthesize phospholipids and neutral lipids for development [41]. A previous study showed that *R*. *similis far-1* expression was higher in the highly pathogenic Rs-C population than in the less pathogenic Rs-P population [9]. Knockdown of *M*. *javanica* FAR-1 in tomato hairy roots reduced infection, while overexpression of the *far* gene could increase nematode infectivity [42]. Suppressing the expression level of *Nb*-*far-1* gene could regulate the growth and development of *N*. *brasiliensis*. This implies that *Nb*-FAR-1 protein may affect the growth and development of *N*. *brasiliensis* by regulating the level of lipids in nematode.

### Knockdown *Nb*-FAR-1 influences the cuticle formation of adult *N*. *brasiliensis*

Interference with *Nb*-*far-1* gene expression only induced the swelling epidermis in L3s, but it caused the crumpled and broken epidermis in adults, accompanied by down-regulated expression in genes for collagen and amino acid transmembrane transport proteins associated with epidermal formation. Collagen is essential for epidermal formation, and interference with *collagen* expression in *C*. *elegans* results in abnormal hypertrophy, dwarfism, and epidermal breakdown in the worm [43]. In *N*. *brasiliensis*, *collagen* genes were significantly low in expression (FPKM value: 0–12) in L3s compared to the relatively high expression in L4s (FPKM value: 117–19819), L5s (FPKM value: 72–18704), and adults (FPKM value: 6–252) of the WT group (S7 Table). In the parasitic stage, L4 larvae, L5 larvae and adult worms experienced rapid growth and dramatic increase in body size, requiring much more collagen proteins for epidermal formation and showing the high expression level of *collagen* genes. Thus, RNAi of *Nb*-*far-1* could not regulate the low expression of *collagen* genes in L3 larvae, but could cause the down-regulated expression of *collagen* genes in adults, which significantly affected adult cuticle formation. Therefore, *N*. *brasiliensis Nb*-FAR-1 may play a role in the formation of nematode cuticle.

## Conclusions

The secretory *Nb*-FAR-1 protein was the abundant protein in *N*. *brasiliensis* ESPs and had an affinity for fatty acids and retinol. Interference with *Nb*-*far-1* gene expression resulted in a decrease in the L3 lipid droplet formation, adult egg-shedding, egg hatchability, larval development, and adult epidermal status. The *N*. *brasiliensis Nb*-FAR-1 protein may be a causative agent in nematode growth, development, and reproduction by affecting the level of lipids.

## Supporting information

S1 Table The location of *far* genes on the chromosomes of *N*. *brasiliensis*. (XLSX)

S2 Table Expression pattern of 12 *N*. *brasiliensis far* genes (FPKM). (XLSX)

S3 Table Mass spectrometry identification of excretory secretions of adult *N*. *brasiliensis in vitro* culture. (XLSX)

S4 Table Transcriptome data of L3s and adults of *N*. *brasiliensis* with RNAi of *Nb*-*far-1* gene. (XLSX)

S5 Table Expression pattern of reproduction-related genes in *N*. *brasiliensis* with *Nb*-*far-1* RNAi and across developmental stages (FPKM). (XLSX)

S6 Table Expression pattern of development-related genes in *N*. *brasiliensis* with *Nb*-*far-1* RNAi and across developmental stages (FPKM). (XLSX)

S7 Table Expression pattern of cuticle-related genes in *N*. *brasiliensis* with *Nb*-*far-1* RNAi and across developmental stages (FPKM). (XLSX)

S1 Fig The analysis of *Nb*-*far-1* gene structure and prokaryotic expression of *N*. *brasiliensis Nb*-*far-1* gene in *E*. *coli*. (A) Gene structure of *Nb*-*far-1* gene. (B) Cloned fragment of *Nb*-*far-1* gene in plasmid pET-28a and prokaryotic expression of *Nb*-*far-1* gene in *E*. *coli*.
(TIF)

S2 Fig Silver staining identification of excretory secretions of adult *N*. *brasiliensis in vitro* culture for 24 h. M: marker.
(TIF)

S3 Fig The binding affinity of *Nb*-FAR-1 protein with DAUDA and retinol and competitive binding of fatty acids with *Nb*-FAR-1 and DAUDA. (A-F) Competition binding effect of different concentrations of unlabeled fatty acids with different carbon chain lengths and number of double bonds on the fluorescence intensity of DAUDA-*Nb*-FAR-1 complex (measured at 380~700 nm). C16:0, palmitic acid; C18:0, stearic acid; C18:1, oleic acid; C18:2, linoleic acid; C20:4, arachidonic acid; C20:5, eicosapentaenoic acid. *Nb*-FAR-1 protein:10 μM, DAUDA:10μM.
(TIF)

S4 Fig DEGs of adult *N*. *brasiliensis* with RNAi of *Nb*-*far-1* gene. (A) Adult_310 group had 199 up-regulated DEGs and 848 down-regulated DEGs compared to Adult_WT group, and 137 up-regulated DEGs and 369 down-regulated DEGs compared to Adult_EV group. (B) Venn diagram of the intersection of DEGs in each group. (C) GO analysis of 506 DEGs between Adult_310 and Adult_EV groups. (D) KEGG analysis of 506 DEGs between Adult_310 and Adult_EV groups.
(TIF)

S5 Fig DEGs of L3 *N*. *brasiliensis* with RNAi of *Nb*-*far-1* gene. (A) L3_310 group had 198 up-regulated DEGs and 561 down-regulated DEGs compared to L3_WT group, and 271 up-regulated DEGs and 119 down-regulated DEGs compared to WT group. (B) Venn diagram of the intersection of DEGs in each group. (C) GO analysis of 390 DEGs between L3_310 and L3_EV groups. (D) KEGG analysis of 390 DEGs between L3_310 and L3_EV groups.
(TIF)

S6 Fig The body weight and the pathological changes of SD rats infected with 2000 L3s of *N*. *brasiliensis*. (A) The body weight of infected rats. (B) H&E staining observation of the pathological changes in the lungs and intestines of infected rats at 12 dpi. Naïve group: normal rats without infection; WT group: rats infected with wide type of L3s; EV group: rats infected with L3s treated with empty virus; *Nb*-*far-1*-310 group: rats infected with L3s treated with LV-*Nb*-*far-1*-310. The value of body weight represents average ± standard deviation.
(TIF)

S7 Fig Effects of LV-mediated *Nb*-*far-1* RNAi on the size of eggs and larvae and the ecdysis of L3s of *N*. *brasiliensis*. (A-C) Effects of *Nb*-*far-1* RNAi on the size of eggs, L1s and L2s. (D) Effects of *Nb*-*far-1* RNAi on the rate of L3s ecdysis. *** *p* < 0.001.
(TIF)

S8 Fig Expression pattern of metabolic DEGs in L3s and adults of *N*. *brasiliensis* with RNAi of *Nb*-*far-1* gene and across developmental stages. (TIF)

S9 Fig SEM observation of epidermal morphology of *N*. *brasiliensis* L3s with RNAi of *Nb*-*far-1* gene. (TIF)
