# Supplementary figures and images for: Nb-FAR-1: A key developmental protein affects lipid droplet accumulation and cuticle formation in Nippostrongylus brasiliensis
Source: PLoS Negl Trop Dis. 2025 Jan 17;19(1):e0012769. doi: 10.1371/journal.pntd.0012769 (PMC11741380; doi:10.1371/journal.pntd.0012769)

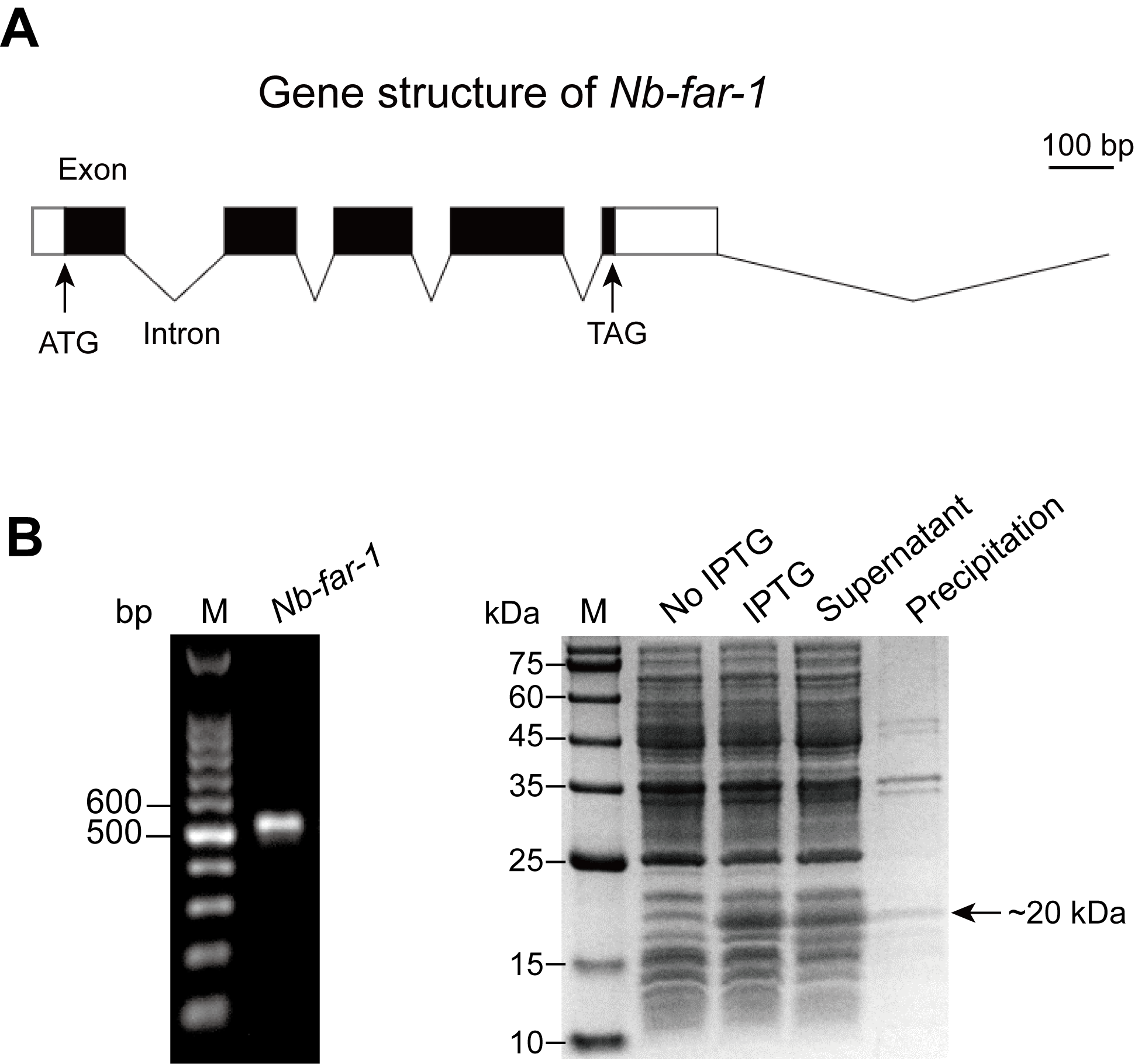

Supplement: S1 Fig — (A) Gene structure of Nb-far-1 gene. (B) Cloned fragment of Nb-far-1 gene in plasmid pET-28a and prokaryotic expression of Nb-far-1 gene in E. coli. (TIF) [file pntd.0012769.s008.tif]

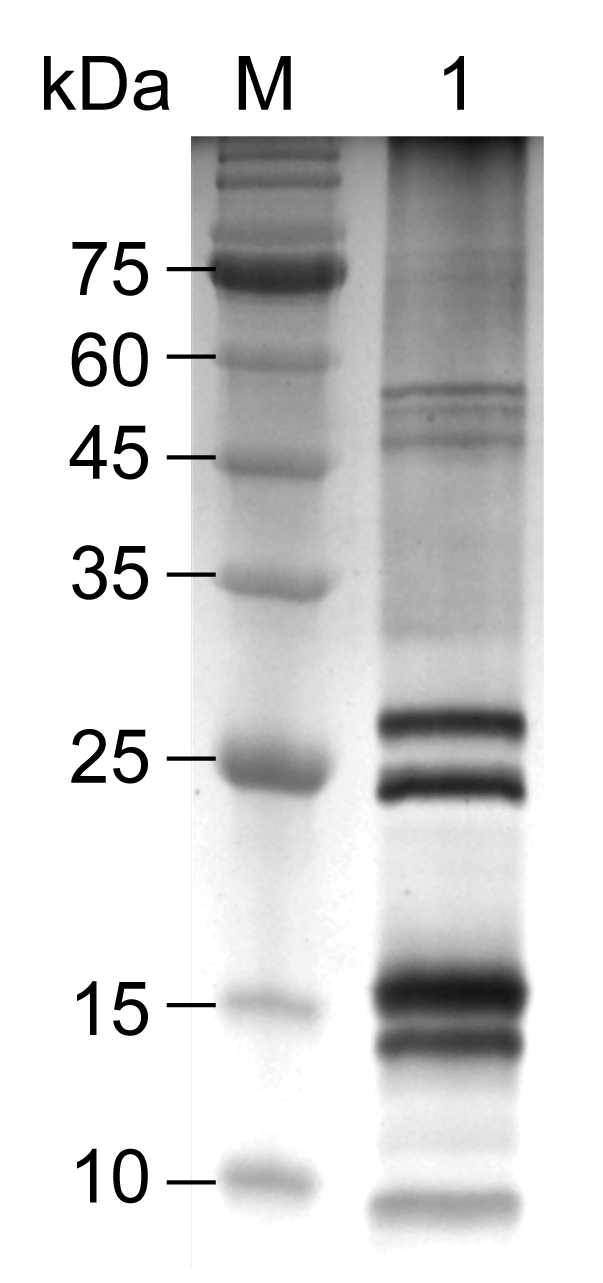

Supplement: S2 Fig — M: marker. (TIF) [file pntd.0012769.s009.tif]

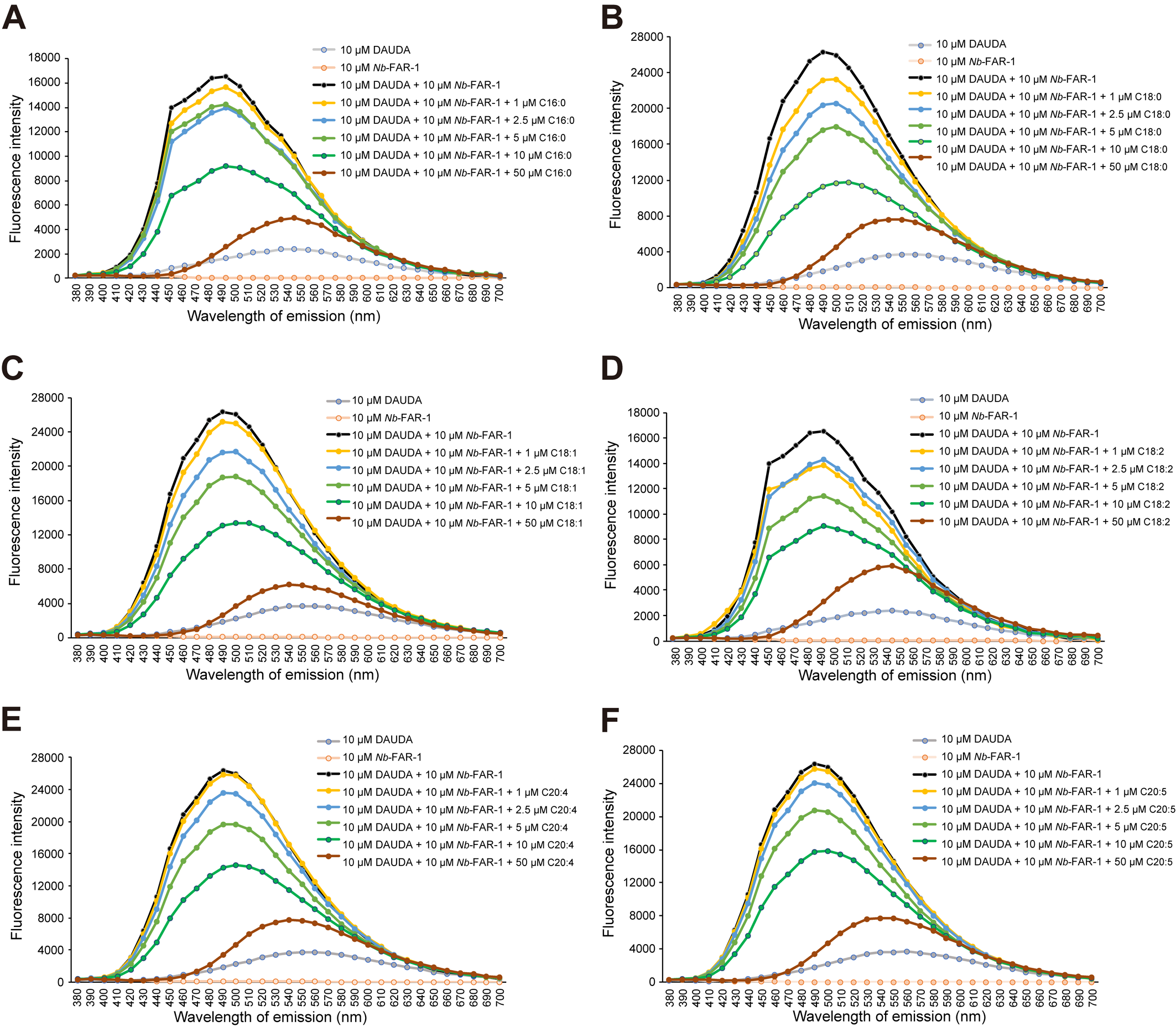

Supplement: S3 Fig — (A-F) Competition binding effect of different concentrations of unlabeled fatty acids with different carbon chain lengths and number of double bonds on the fluorescence intensity of DAUDA-Nb-FAR-1 complex (measured at 380~700 nm). C16:0, palmitic acid; C18:0, stearic acid; C18:1, oleic acid; C18:2, linoleic acid; C20:4, arachidonic acid; C20:5, eicosapentaenoic acid. Nb-FAR-1 protein:10 μM, DAUDA:10μM. (TIF) [file pntd.0012769.s010.tif]

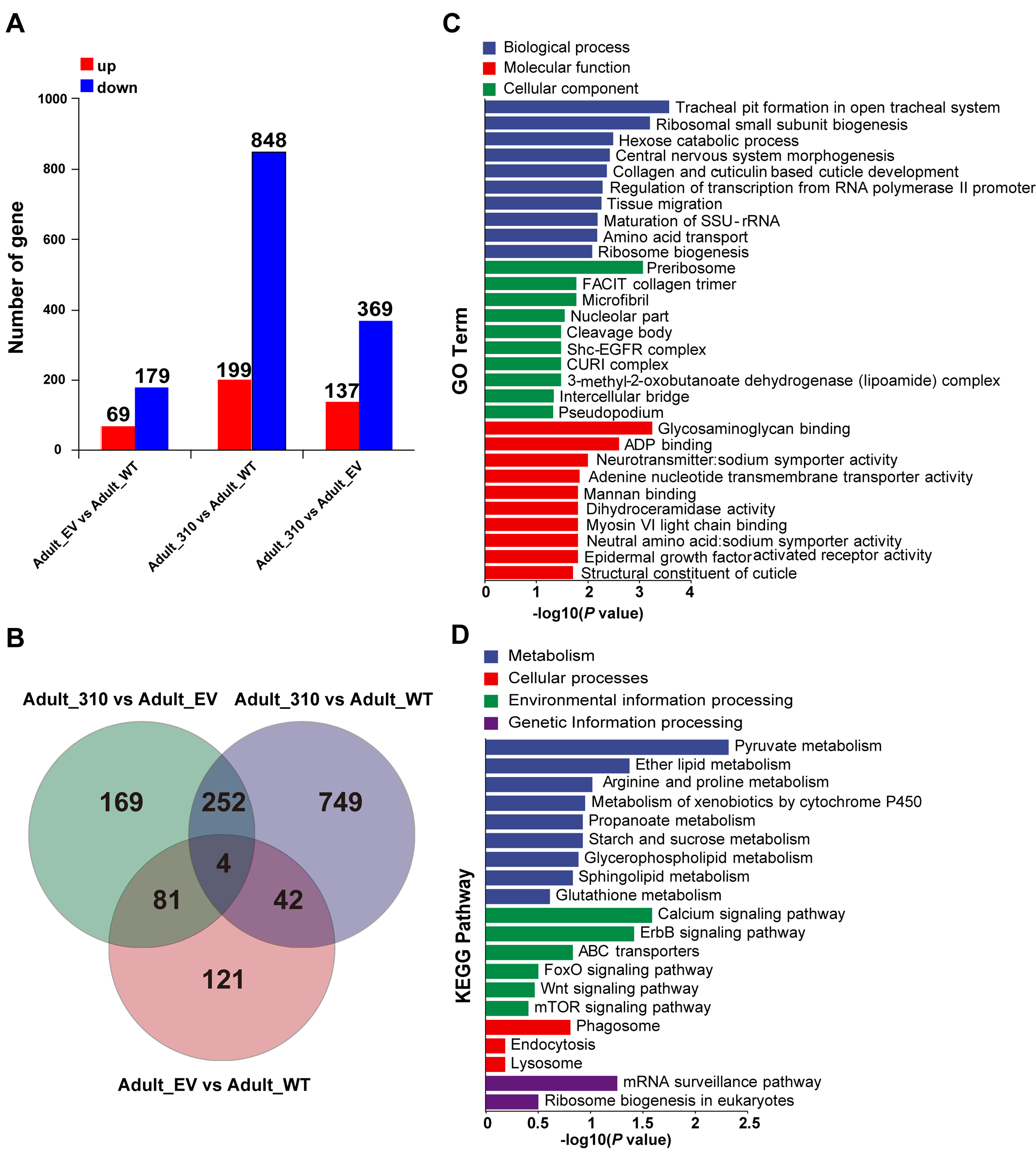

Supplement: S4 Fig — (A) Adult_310 group had 199 up-regulated DEGs and 848 down-regulated DEGs compared to Adult_WT group, and 137 up-regulated DEGs and 369 down-regulated DEGs compared to Adult_EV group. (B) Venn diagram of the intersection of DEGs in each group. (C) GO analysis of 506 DEGs between Adult_310 and Adult_EV groups. (D) KEGG analysis of 506 DEGs between Adult_310 and Adult_EV groups. (TIF) [file pntd.0012769.s011.tif]

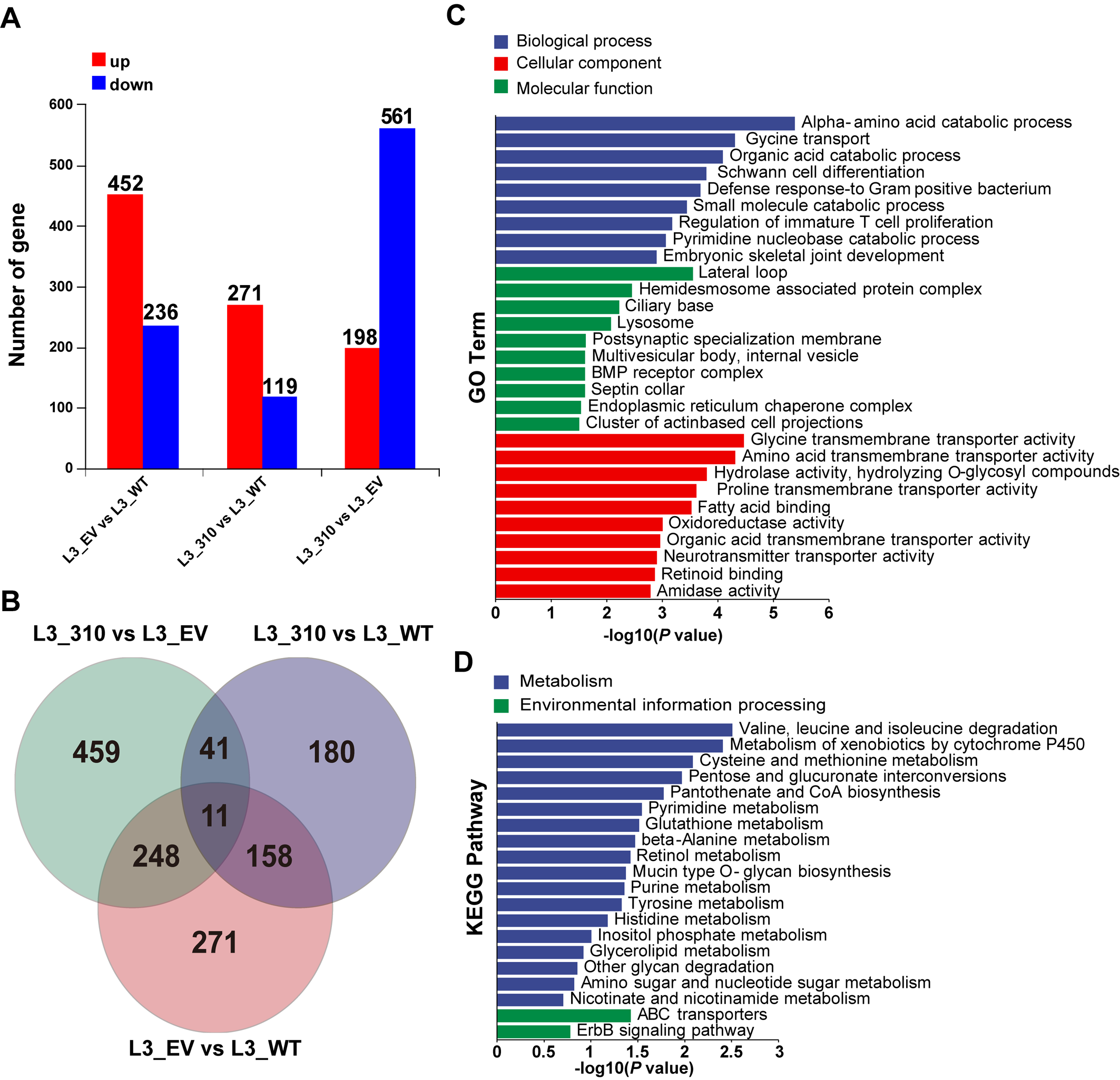

Supplement: S5 Fig — (A) L3_310 group had 198 up-regulated DEGs and 561 down-regulated DEGs compared to L3_WT group, and 271 up-regulated DEGs and 119 down-regulated DEGs compared to WT group. (B) Venn diagram of the intersection of DEGs in each group. (C) GO analysis of 390 DEGs between L3_310 and L3_EV groups. (D) KEGG analysis of 390 DEGs between L3_310 and L3_EV groups. (TIF) [file pntd.0012769.s012.tif]

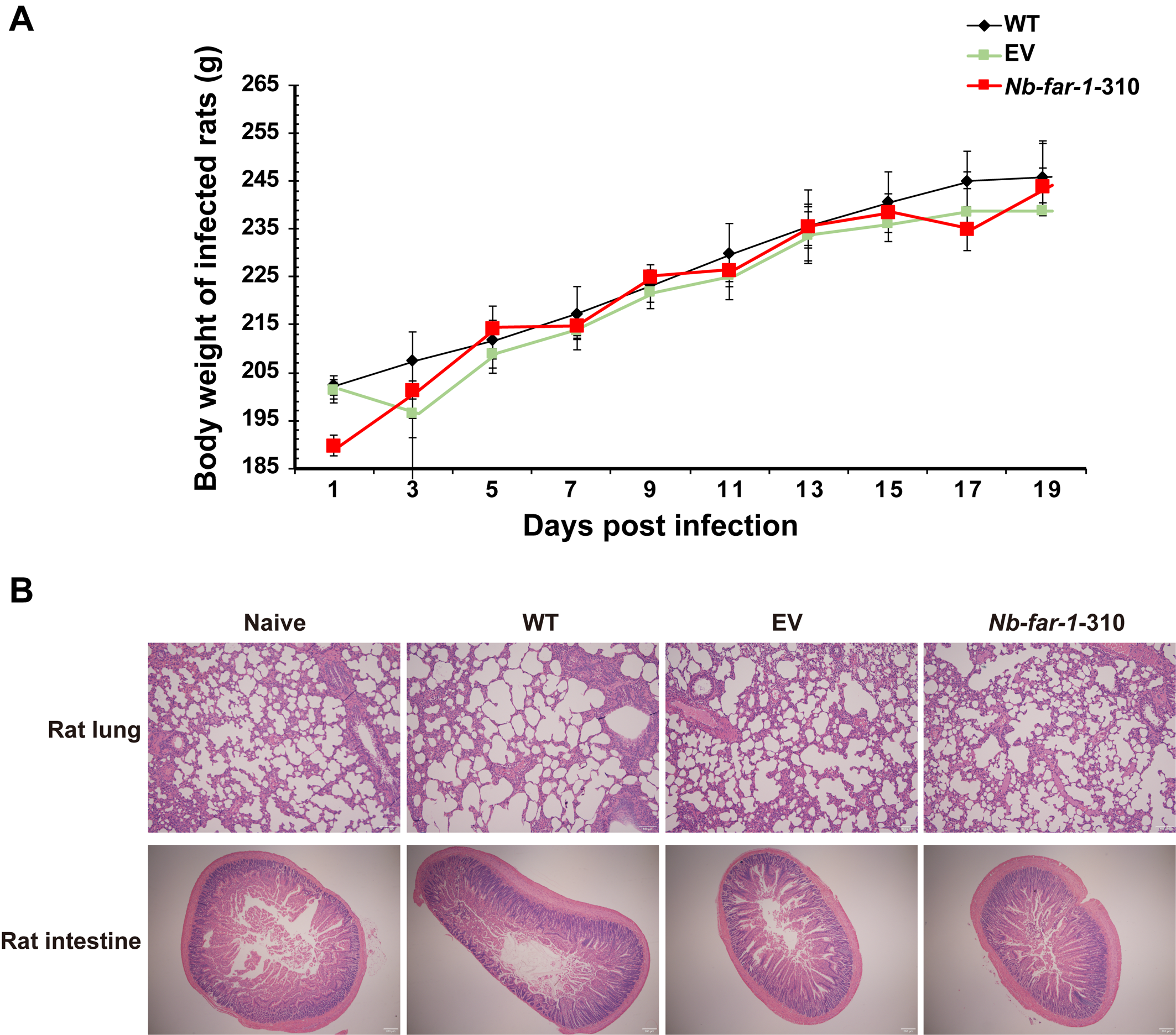

Supplement: S6 Fig — (A) The body weight of infected rats. (B) H&E staining observation of the pathological changes in the lungs and intestines of infected rats at 12 dpi. Naïve group: normal rats without infection; WT group: rats infected with wide type of L3s; EV group: rats infected with L3s treated with empty virus; Nb-far-1-310 group: rats infected with L3s treated with LV-Nb-far-1-310. The value of body weight represents average ± standard deviation. (TIF) [file pntd.0012769.s013.tif]

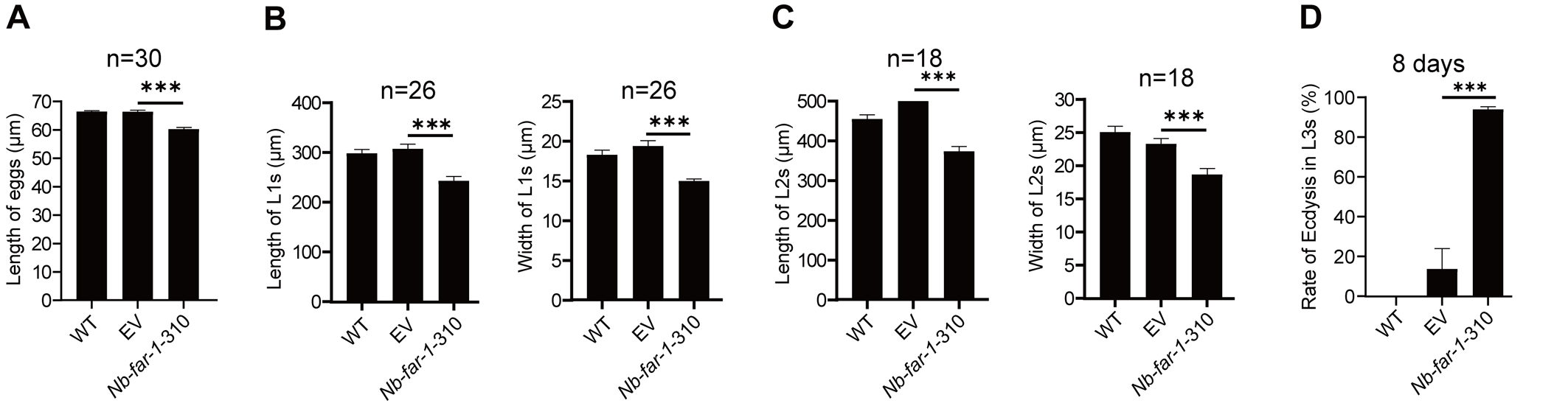

Supplement: S7 Fig — (A-C) Effects of Nb-far-1 RNAi on the size of eggs, L1s and L2s. (D) Effects of Nb-far-1 RNAi on the rate of L3s ecdysis. *** p < 0.001. (TIF) [file pntd.0012769.s014.tif]

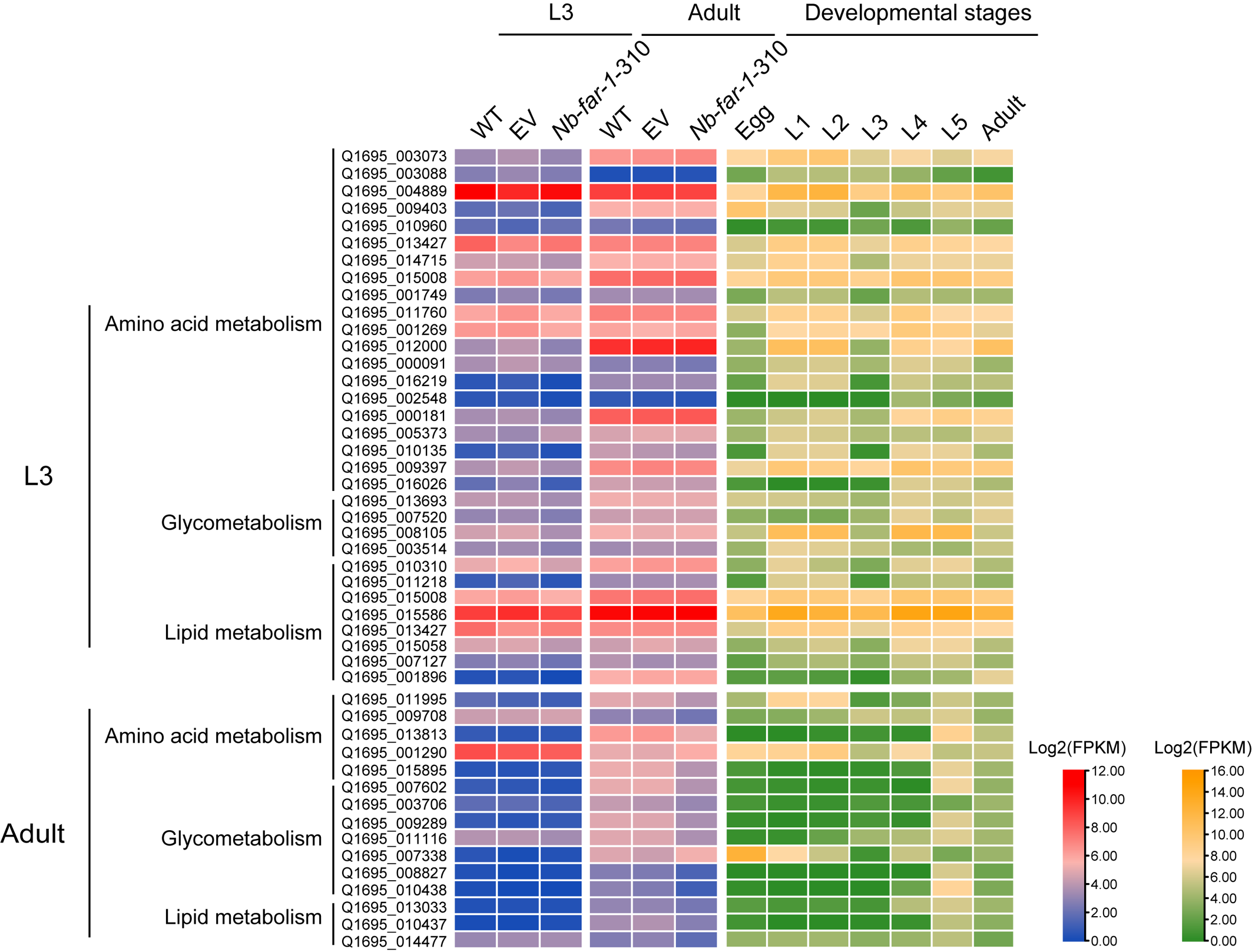

Supplement: S8 Fig — (TIF) [file pntd.0012769.s015.tif]

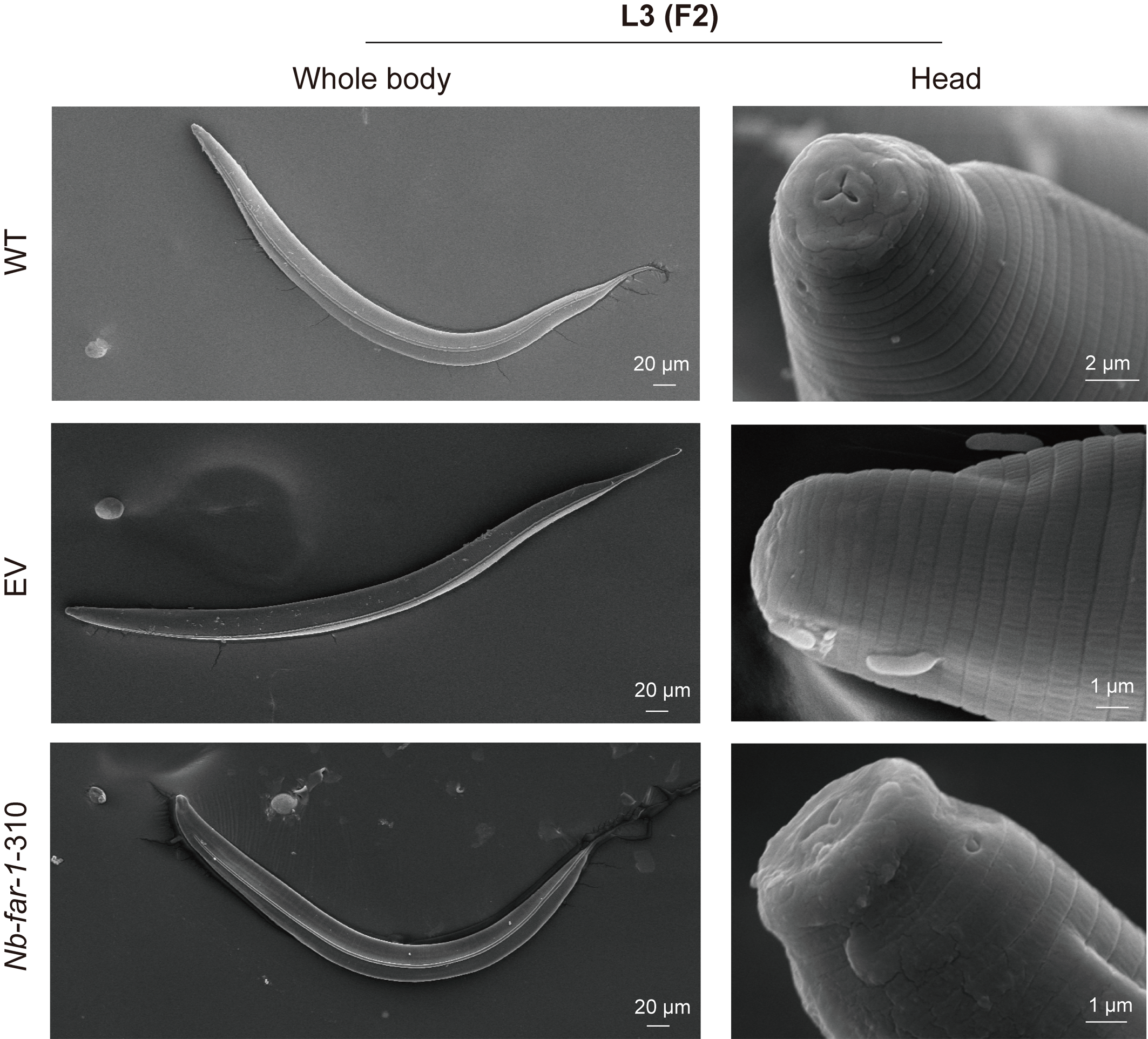

Supplement: S9 Fig — (TIF) [file pntd.0012769.s016.tif]
